# Computational framework for fusing eye movements and spoken narratives for image annotation

**DOI:** 10.1167/jov.20.7.13

**Published:** 2020-07-17

**Authors:** Preethi Vaidyanathan, Emily Prud'hommeaux, Cecilia O. Alm, Jeff B. Pelz

**Affiliations:** Eyegaze Inc., Fairfax, VA, USA; Computer Science Department, Boston College, Boston, MA, USA; College of Liberal Arts, Rochester Institute of Technology, Rochester, NY, USA; Chester F. Carlson Center for Imaging Science, Rochester Institute of Technology, Rochester, NY, USA

**Keywords:** multimodal fusion, eye movements, spoken descriptions, gaze, bitext alignment, machine translation, computer vision, image annotation

## Abstract

Despite many recent advances in the field of computer vision, there remains a disconnect between how computers process images and how humans understand them. To begin to bridge this gap, we propose a framework that integrates human-elicited gaze and spoken language to label perceptually important regions in an image. Our work relies on the notion that gaze and spoken narratives can jointly model how humans inspect and analyze images. Using an unsupervised bitext alignment algorithm originally developed for machine translation, we create meaningful mappings between participants’ eye movements over an image and their spoken descriptions of that image. The resulting multimodal alignments are then used to annotate image regions with linguistic labels. The accuracy of these labels exceeds that of baseline alignments obtained using purely temporal correspondence between fixations and words. We also find differences in system performances when identifying image regions using clustering methods that rely on gaze information rather than image features. The alignments produced by our framework can be used to create a database of low-level image features and high-level semantic annotations corresponding to perceptually important image regions. The framework can potentially be applied to any multimodal data stream and to any visual domain. To this end, we provide the research community with access to the computational framework.

## Introduction

The use of digital images range from personal photos and social media to more complex applications in education and medicine. In addition to serving as a means for documenting events and capturing memories, digital images can help facilitate decision making. Doctors use medical images to help diagnose and determine the treatment of diseases, and emergency response is often guided by imagery available from the scene. Intelligent computers should be capable of making inferences about where people look and what they say about the things they see. We believe computers would benefit by acquiring and using learned associations. This is known as *semantic image annotation*. When applied to identify regions in images, it is called *semantic image region annotation*. A system capable of accurate semantic image region annotation would be able to provide a user useful and detailed information about an image. This work integrates gaze and linguistic information indicating ‘what people look at’ and ‘what people say,’ to identify the objects and their corresponding names or labels in images. The data we collected (Vaidyanathan et al., 2018) which has been released for research purposes and the code we developed for the framework (released in this work), allowed us to explore the following research questions:

RQ1: When a person views and describes an image, what relationship, if any, exists between the moment of fixation on an object and the moment the person utters the word or phrase to name that object?
RQ2: Can co-captured gaze and speech data be integrated automatically in order to identify and quantify this relationship?
RQ3: Can the discovered relationship or relationships be used to extract meaningful, accurate information about the objects in an image?

The dataset can also be useful for scholars who wish to study language production during scene-viewing tasks, including the interaction between word complexity and frequency and gaze behavior. Further, this multimodal dataset can be used in studies of affective visual or linguistic computing tasks. The multimodal framework we propose establishes the utility of combining gaze and spoken descriptions and highlights the potential of additionally considering multiple modalities in studies of human perception (e.g., facial expression, pulse rate, galvanic skin response). The framework we have developed for the purpose of semantic image region annotation could also be used for real-world computer vision applications, such as interactively annotating regions of interest in works of art on display in a museum.

**Figure 1.**
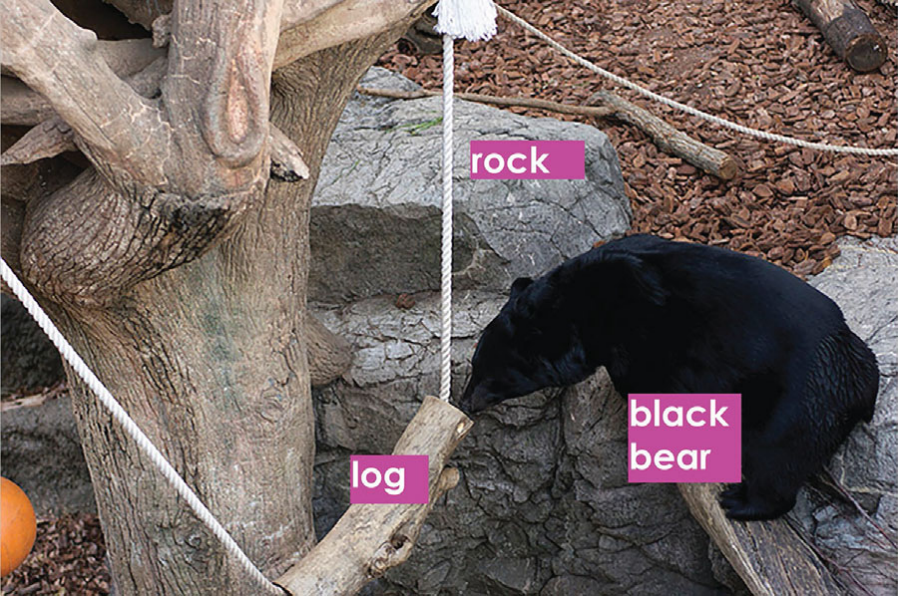
Example illustrating the concept of semantic image region annotation. The process involves identifying and segmenting perceptually meaningful regions in an image and labeling them appropriately. Image credit: “Creative Commons Asian black bear” by Taro Sako, used under CC BY-NC 2.0. Text overlaid on original.

Automatic semantic image region annotation plays a key role in developing sophisticated image-based information systems but is a difficult and long-standing problem (Smeulders et al., 2000; Zhang et al., 2012; Karpathy & Fei-Fei, 2015). An illustration of semantic image region annotation where regions in an image are descriptively labeled with appropriate words is shown in Figure 1. Although the entire image in Figure 1 could be annotated as, for example, *bear playing with a log*, it is intuitive to annotate objects or subregions with labels such as *bear* and *log*. These detailed annotations for image regions can assist in important applications such as image retrieval where the user could be searching for images of bears or visual question-answering where the user could be asking what the bear is playing with. Further, relationships between annotated regions could also be inferred, for example, the bear is sniffing the log. High-level cognitive processing and experience enable humans to process images at a semantic level that remains difficult for a computer (Shanteau, 1992; Goldstone, 1998; Zhu et al., 2016; Zitnick et al., 2016; Tavakoli et al., 2017).

Gaze locations distributed across an image can act as pointers and reveal perceptually important regions and their relation to one another from the perspective of an observer. Spoken language is the most natural and convenient instrument of expression for humans to communicate their understanding of and reasoning about images. In this case co-captured image descriptions convey relevant meaning, particularly special knowledge and experience that the human observers possess. An important aspect of this work lies in the integration of human observers’ perceptual and conceptual knowledge using natural language processing (NLP) methods to annotate images.

People often have the intuition that when they look at an object and mention its name, they do so simultaneously. However, research in sentence production has shown that there is a variable amount of time between when a person looks at an object and when they name it aloud (Meyer et al., 1998; van der Meulen, 2003; Griffin, 2004; Vaidyanathan et al., 2012). Therefore, even when visual and linguistic information is co-captured we cannot assume that a fixation on a region will occur simultaneously with the verbal naming of the region. This lag, which can vary in length, demands more sophisticated methods.

The bitext word alignment method (Brown et al., 1993; Liang et al., 2006), widely used in statistical machine translation, aligns each word in a sentence in one language with the word or words in a parallel sentence in a second language that are most likely to have the same meaning. In this work, the unimodal parallel sentences are replaced by fixations and spoken descriptions; the fixation locations on images are analyzed as *visual units* that encode visual regions while the spoken descriptions about the images contain the parallel *linguistic units*. Prior work confirms the usefulness of associating words and sentences with images, objects and image regions, and videos particularly in interpreting images, generating image captions, coreference resolution in text, and natural language descriptions for videos (Forsyth et al., 2009; Kuznetsova et al., 2013; Kong et al., 2014; Thomason et al., 2014). Many of these works rely on written descriptions of general-domain images, making the framework difficult to translate to domain-specific images. Since these works need written descriptions, it is difficult and laborious to translate them to domains like dermatology where experts are required. However, our framework can be applied to any image domain (Vaidyanathan et al., 2016). Perceptual and conceptual information is combined via the integration of gaze and narratives to advance annotation of image regions (Vaidyanathan, 2017).

**Figure 2.**
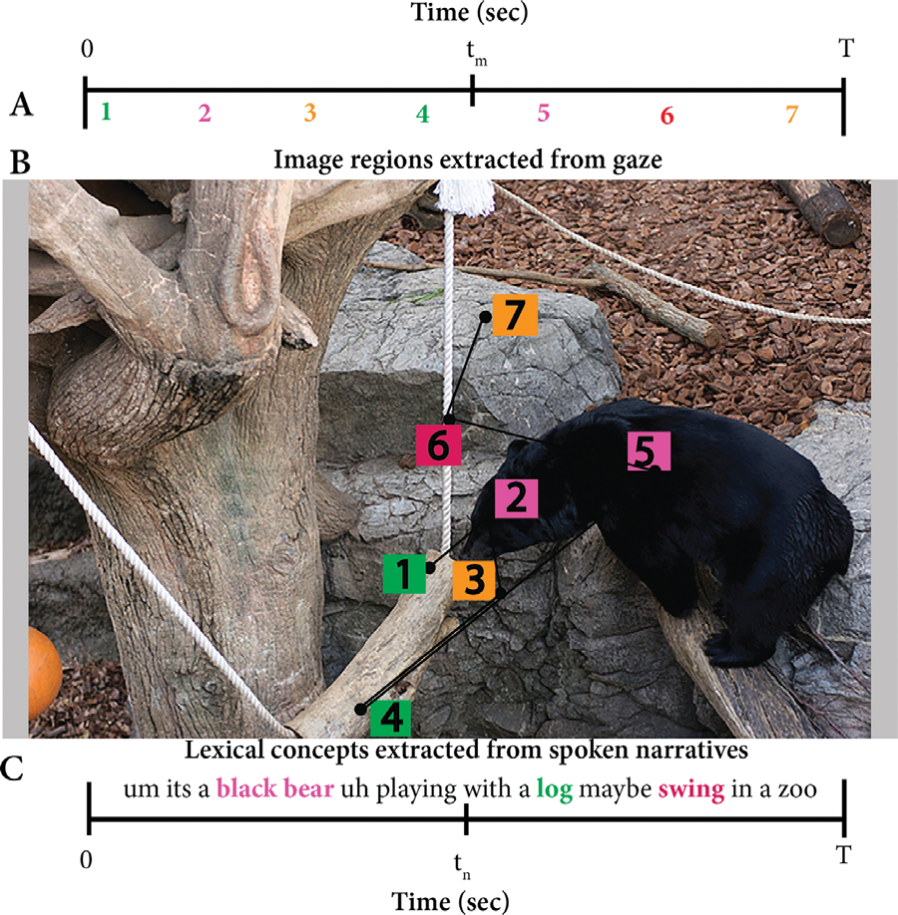
Panels A and C show the gaze fixation locations extracted from eye movements and lexical concepts (labels) obtained from spoken narratives, respectively, over a common time scale. This hypothetical example shows that the data collection session for this image took T seconds. Panel B shows the seven image regions that were looked at by the observer in the original image. The proposed algorithm will align words such as *bear* and *log* with corresponding regions, using the bitext alignment technique within the discussed multimodal framework. Image credit: “Creative Commons Asian black bear” by Taro Sako, used under CC BY-NC 2.0. Scanpath overlaid on original.

The aim of this study is to understand and encode important image information by semantically annotating important regions of an image with natural language descriptors as shown Figure 1. The framework uses gaze locations on images together with words uttered by observers to learn perceptually important image regions and the corresponding linguistic descriptors, as shown in Figure 2. The study also asserts that the combination of perceptual information (via eye movements) and more naturally obtained conceptual information (via spoken narratives) contributes to the understanding of an image.

The four main contributions of this work are as follows:

(1) Demonstrate that human-elicited gaze and narratives jointly provide information that if considered separately would be insufficient to understand how humans perform image inspection and description tasks.
(2) Exemplify the applicability of the visual-linguistic alignment framework by comprehensively using and evaluating it with a general-domain image dataset.
(3) Compare the performance of different image region segmentation techniques used to identify the visual units in order to determine their strengths and weaknesses.
(4) Provide the research community with access to the framework,^1^ which can be extended to integrate modalities other than those discussed in this work.

## Related work

### Challenges in image annotation

The goal of this work is to automatically annotate images through the integration of users’ cognitive perceptual (gaze) and conceptual (spoken language) information with information contained in the images. Treisman and Gelade (1980) proposed that processing of image information is a dynamic interaction between bottom-up low-level image information pieces and top-down user-driven directed processes. In spite of the proposed integration theory, for a long time image annotation algorithms were built solely on low-level features such as color and texture to perform segmentation and retrieval (Saber et al., 1996; Shi & Malik, 2000). Algorithms employing these low-level features succeeded in capturing basic statistics of natural scenes (Fei-Fei & Perona, 2005), identifying faces (Viola & Jones, 2004), or segmenting single objects in a scene (Kumar et al., 2010; Jaber & Saber, 2010), but they were unable to deal with multiple objects in the scene, complexity of domain-related images, and other high-level processing tasks. For example, while the bottom-up methods helped in automatic detection and segmentation of objects in a scene, they did not provide the relationship between these objects or the contextual meaning of the scene (Li et al., 2009). Recently researchers have had some success with generating image descriptions and semantic labeling of general-domain images (Kong et al., 2014; Karpathy & Fei-Fei, 2015; Yatskar et al., 2016; Vasudevan et al., 2018b, 2018a; Anderson et al., 2018; Gygli & Ferrari, 2018). However, it is not clear if their techniques would easily translate to complex domains involving experts.

To bridge the semantic gap, Duygulu et al. (2002) proposed the use of machine translation to combine image content with the accompanying text for object recognition. Following this, other researchers proposed several integrating techniques using different mathematical approaches such as Bayesian methods, latent Dirichlet allocation and latent semantic analysis methods (Barnard et al., 2003; Li & Wang, 2003; Berg et al., 2004b, 2004a). Similarly, researchers proposed the use of deep learning to combine text and images for image annotation (Karpathy & Fei-Fei, 2015; Vinyals et al., 2014), as well as unsupervised alignment to align text instructions with video segments (Naim et al., 2014). Johnson et al. (2015) suggested the use of neighboring test images and their annotations to disambiguate and annotate otherwise ambiguous images. These approaches bridge the semantic gap to a certain extent by bringing in multimodal information through images and text. However they do not involve human expertise or cognitive knowledge encoded via speech or gaze data that is important to capture the semantics of images in complex domains (Tourassi et al., 2013; Kumar et al., 2016; Qu & Chai, 2008) extended the idea of using multimodal data by using more natural speech and eye gaze than previous work, but their application scenario was a 3D simulated scene and did not involve real-life challenges such as occlusion.

### Importance of capturing perceptual and conceptual information

Fixations can be considered pointers to perceptually important regions of an image while spoken narratives can reveal conceptual elements associated with those regions. Capturing perceptual and conceptual information relevant to the image processing system's end user's goal is of paramount importance for improving the annotation of images. Image-information systems must be reliable enough to assist in goal-oriented performance (Müller et al., 2004). End users typically do no merely seek images or regions that have similar low-level features such as color or texture but they instead want to locate, classify, or segment an image based on high-level reasoning features. Studies have found that perceptual and conceptual information help a user formulate more specific and comprehensive descriptions of images and these correlate with the user's ability to express their information needs (Goldstone, 1998; Krupinski, 2000; Vakkari, 2002; Hoffman & Fiore, 2007).

Researchers have used various knowledge-elicitation methods to capture human users’ expertise. One of the most common methods is interviewing and asking participants to describe the decision making process through the think-aloud protocol. One problem with this method is that it will only produce what an expert can verbalize as an answer to the particular question (Shadbolt & Smart, 2015). It also requires the expert to perform a secondary task in parallel with the primary task. Any non-verbalizable information, such as where these experts look in the image, is lost, and there is a risk that the expert may not verbalize freely when they are uncertain or confused. Another widely used technique is to ask the experts to manually mark important regions in images (Shyu et al., 1999; Wang et al., 2012b). The drawback with this technique is the loss of any information pertaining to how the expert arrived at that decision. This work uses eye movements and spoken language as they are non-invasive and more natural tools that enable us to draw out the tacit perceptual and conceptual information of humans.

### Need to integrate eye movements and spoken narratives

Experiments have shown that eye movements are closely time-locked with human language processing (Just & Carpenter, 1976; Ferreira & Tanenhaus, 2007; Griffin, 2004). In the field of psycholinguistics, eye movements have been used as a tool to understand language processing. Similarly, eye movement researchers have incorporated linguistic input into their studies. Just and Carpenter (1980) described how measures like fixation duration changed depending on the linguistic characteristics of the text being read. Soon several researchers began using eye movements as a tool to reveal the way written language is processed (Frazier & Rayner, 1982; Heller, 1988; Pollatsek et al., 1993; Rayner, 1998). Some researchers studied language comprehension through the use of eye movements (Tanenhaus et al., 1995; Dahan et al., 2001; Spivey et al., 2002; Richardson & Dale, 2005; Cooper, 1974). They revealed that it was possible to investigate how people understand spoken language by measuring people's eye movements while listening to verbal commands and executing them. Richardson and Dale (2005) conducted a study to understand the coupling between speakers and listeners, reporting that the interlocutors’ eye movements were closely time-locked. Kaiser and Trueswell (2008) showed that eye movements can be used to understand the stages of language comprehension such as hearing a command, interpreting it, and engaging in resolving and executing commands. This work revealed that a relation between cognition, vision, and language exists and that by integrating eye movements and spoken narratives, complex cognitive tasks can be understood. Meyer et al. (1998) investigated eye movements and object naming and found that fixations on objects were delayed for lexical processing; when required to name the objects, gaze did not move to the next object until the phonological form of the current object was retrieved. Meyer et al. (1998) also observed that mean viewing time for speakers was significantly longer for objects with low frequency names than with high frequency names. In another study, van der Meulen (2003) demonstrated that participants fixated the objects to be named in the order of mention and once just before naming or describing using an adjective. This indicates that speech is performed in an incremental fashion, i.e. speakers keep looking at the current object until they find the words for the object before moving to the next object.

The growing interest in this multimodal field motivated Griffin and Bock (2000) to study the temporal relation between event apprehension, sentence formulation, and speech execution. Their study involved an ‘agent’ and a ‘patient’ (the object being acted on) involved in a simple event. Participants were asked to inspect and describe the event in one sentence without pronouns. They analyzed the timing of specific fixations to agents and patients and found that speakers’ eye movements were guided by an overall understanding of the event/scene rather than by the salience of the individual objects in it. The distribution of fixation times anticipated the order of mention regardless of sentence structure, partly confirming the findings by van der Meulen (2003). We believe that as the complexity of the image and task increases, fixations on objects may not follow the same order in which objects were named. This indicates we need methods like word alignment to handle repetitive or unimportant words and fixations as well as one-to-many and many-to-one relationships. They also found that when speaking extemporaneously, speakers began fixating elements less than a second before naming them, suggesting that people spend some time looking at objects prior to naming them (Griffin & Bock, 2000; Griffin, 2004). More recently, a study was conducted to understand how complex noun phrases are produced and if the production process was similar to that of simple noun phrases (Shao et al., 2013).

The above findings indicate that vision and language are tightly integrated. Several researchers have investigated methods to combine the two cognitive modalities to understand semantic processing (Badler, 1975; Waltz, 1980; Herzog & Wazinski, 1994; Srihari, 1995). Deb Roy proposed a technique to integrate vision and language elicited from infants using a mutual information model (Roy, 2000; Roy & Pentland, 2002). In the last decade, several researchers began studying the multimodal integration problem in relation to sentence prediction and object naming in scenic images (Coco & Keller, 2012; Clarke et al., 2013; Yun et al., 2013a, 2013b). Although there is some relationship between the timing of eye movements and spoken narratives, an exact or fixed-delay temporal match indicating that a fixation on a region will occur simultaneously or after a fixed time interval with the verbal naming of the region cannot be assumed. Holsanova (2006) studied the interaction of vision and language over time by investigating the dynamics of picture viewing and picture description. Her research revealed that correspondence between the spoken words and the objects in the scene could be of different types, for example, one-to-one or many-to-one. These findings partly confirm hypotheses such as the existence of a temporal relationship between when objects are fixated and when their names are uttered, but lack any quantitative consistency that would enable modeling that could be used in automated systems. Therefore, we need to use other techniques such as bitext alignment.

There has been a large body of research on using machine learning to identify objects or regions in images using human-generated keywords or captions. Duygulu et al. (2002) investigated a method to automatically recognize and annotate objects in scenes. They segmented images into regions and clustered them into region types that they referred to as *blobs*. Then, an expectation-maximization method was used to learn the mapping between the blobs and the keywords for a given image. However, the image regions or blobs and keywords were obtained using image segmentation methods and a large vocabulary from captions without any human-elicited eye movements and spoken narratives. A similar technique was used by other scholars to automatically match words to the corresponding pictures (Barnard et al., 2003), faces in pictures to names (Berg et al., 2004a, 2004b), and natural language instructions to video frames for a particular task (Naim et al., 2014). However, none of these works incorporate gaze information.

Yu and Ballard (2004b) seem to be the first to explore how word alignment methods could be extended to the challenging task of grounding spoken language in visual perception. Similar to our work, they transcribed the audio and extracted nouns as object names. For the perceptual representation of objects, Yu and Ballard (2004b) segmented the objects in the video using gaze data. Further, these objects were represented using multidimensional color and shape features. The multimodal data consisting of words and objects was then integrated using IBM Model 2 (Brown et al., 1993; Liang et al., 2006), a non Hidden Markov Model-based word alignment method commonly used in machine translation, to learn correspondences. In their extended work, they combined scene video, participant's gaze, head motion, and object names obtained from verbal narratives while performing simple everyday tasks, such as stapling printed papers, to annotate objects and categorize action scenes in video (Yu & Ballard, 2004a). Their work provides a good understanding of how multimodal data can be combined for a video annotation task. However, their work involved only six (Yu & Ballard, 2004a) and nine (Yu & Ballard, 2004b) participants and three simple video stimuli. Primarily, Yu and Ballard explored object annotation with images that had uniform backgrounds and consisted of distinct objects that were easy to segment from the background. Also, their work did not provide a clear evaluation and baseline comparison. Qu and Chai (2008) collected gaze data and spoken responses for computer-generated videos involving 3D objects in a room scene. Participants were asked various questions about the decoration of the 3D simulated room (e.g. *describe the left wall, what do you dislike about this room*.) They proposed a modified IBM Model 2 (Brown et al., 1993) to integrate gaze information and spoken language to help interpret unexpected user language inputs in conversational systems. The use of a simulated 3D room scene does not capture the challenges such as clutter and ambiguity that come with natural images. Similarly, use of question-answering method only provides us with the subject's final answer and may not capture all the elements of gaze and speech leading up to the final answer. Motivated by the work of Yu and Ballard (2004b), we investigate multimodal image region annotation with images that do not have uniform background and consist of multiple objects in images that are challenging to segment. We explore the annotation task using a dataset consisting of general-domain images and provide baseline comparisons.

## Multimodal data collection

**Figure 3.**
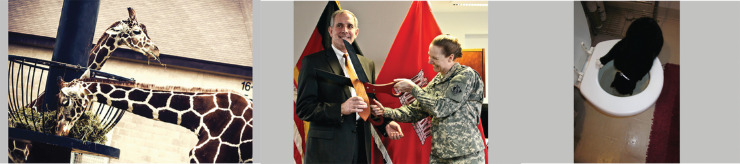
Example images from mscoco used in the data collection process. The images vary in number of objects, scale, lighting, and resolution posing challenges to the alignment framework. Image credits from left: “Giraffes” by Garret Voight, used under CC BY-NC 2.0, “USACE division visit to Europe District coincides with German Fasching celebrations” by U.S. Army Corps of Engineers Europe District, used under CC BY 2.0, “Fresh Water in the House” by Megan, used under CC BY-NC 2.0.

In this section, we describe our multimodal Spoken Narratives And Gaze (snag) dataset (Vaidyanathan et al., 2018) that is used to evaluate the proposed framework. This dataset contains eye movements and spoken narratives co-captured from participants while viewing general domain images (Figure 3) and has been released^2^ to the research community. Coco and Keller (2010) have released a similar dataset; however, in their experiment they showed participants a cue word before each image and instructed them to use the cue word in the description. Recently, van Miltenburg et al. (2018) released a dataset that contains co-captured gaze and spoken Dutch descriptions for images that do not necessarily contain any action. Our dataset is different because it involves images depicting an event and do not involve any cue words that need to be used by participants. Additionally, we use the master-apprentice method to elicit rich descriptions.

Our alignment-annotation framework consists of the following four major steps:

(1) **Collecting multimodal data:** In this step, we collect multimodal data, specifically raw speech and gaze data that are subsequently processed to obtain transcriptions and fixations, respectively.
(2) **Collecting and retrieving units of analysis:** In this step, we extract the units of analysis namely linguistic units from the transcripts and visual units from the fixations.
(3) **Multimodal bitext alignment:** These extracted units of analysis are then fed into the bitext alignment algorithm where they are aligned.
(4) **Labeling the image regions:** The output from the multimodal aligner is used to label image regions.

**Participants:** Our institutional review board-approved data collection involved 40 subjects ranging in age from 18 to 25 years. To ensure reliable automatic speech recognition (ASR) transcription and a consistent vocabulary, only native speakers of American English were selected as participants. Participants were recruited campus-wide from the Rochester Institute of Technology. We used an adapted Master-Apprentice (Beyer & Holtzblatt, 1997) data collection method to elicit more details from observers in a natural context compared to the traditional think-aloud method. Prior studies (Lloyd & Dykes, 2011; Gurses et al., 2012) have shown that, when human experts are placed in a teaching mode, they provide more information and richer detail than when they are in a think-aloud mode. For this reason, we ask our participants to take on the role of a master whose task is to describe and explain the image to the examiner, who acts as the apprentice. We instructed participants to *describe the action in the images and tell the experimenter what is happening* and say *next* when they were done to move to the next image. Although in some contexts the act of viewing and describing simultaneously might seem unnatural, we note that it is commonplace in many settings. Radiologists, for example, routinely dictate descriptions of radiologic images for their reports, military personnel and journalists describe their visual environments to remote collaborators and listeners, museum visitors discuss works of art while viewing them, and caregivers comment on the contents of picture books or interesting things they see when interacting with babies and toddlers.

### Stimuli

We showed participants 100 general-domain images selected from the Microsoft Common Objects in Context (mscoco) open-source dataset (Lin et al., 2014) which consists of more than 300,000 images. The images represent complex everyday scenes containing common objects and people. For our dataset, the first author selected images that typically depicted an event. Example images are shown in Figure 3. The images were presented to the participant on a 22-inch LCD monitor (1680 × 1050 pixels) located at a viewing distance of approximately 68 cm. At 68 cm, the full display subtends 38° × 22° of visual angle.

### Gaze and verbal data

**Figure 4.**
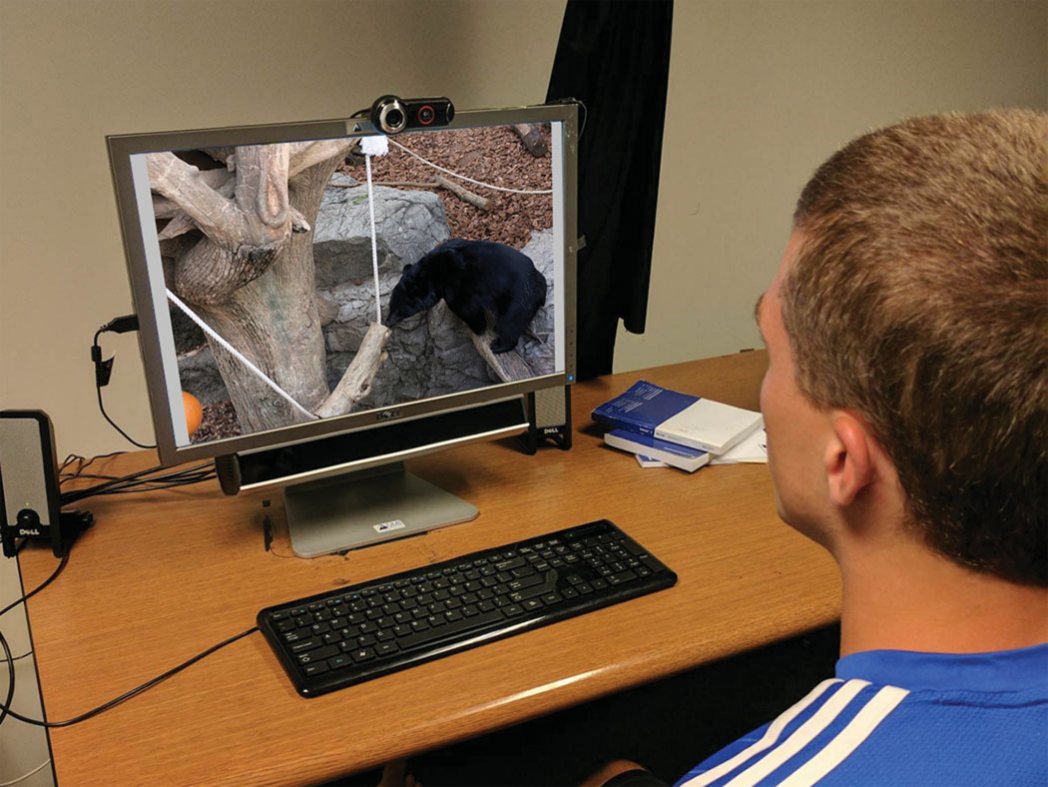
Data collection set-up: the SMI eye-tracker was positioned underneath the stimulus display. The participant wore a lapel microphone connected to a TASCAM recorder that captures the spoken descriptions. The task requires the participant to describe the action in the image to the experimenter. Inset image credit: “Creative Commons Asian black bear” by Taro Sako, used under CC BY-NC 2.0. Original modified to fit as inset.

Eye movement data was collected using a SensoMotoric Instruments (SMI) RED 250Hz eye-tracker attached to a display as shown in Figure 4. The SMI is a nonintrusive remote eye-tracker with a reported accuracy of 0.5°. We used a double computer set-up with one computer used to run the SMI software iViewX gaze tracking system and Experiment Center 2.3 and the other used for stimulus presentation. Each stimulus was followed by a blank gray slide to minimize the effect of gaze from the prior stimulus. The blank gray slide was followed by a test slide with a small, visible target at the center with an invisible trigger area of interest. Using the test slide, we could measure any drift between the location of the target at the center and the predicted gaze location over time that may have occurred owing to the participants’ movements. Each participant performed a nine-point calibration at the beginning of their trial, followed by a validation after every 10 images and recalibration if their validation error was more than one degree. We used a TASCAM DR-100MKII audio recorder with a lapel microphone to collect the speech recordings. Participants were given a mandatory break after 50 images and otherwise smaller breaks if needed to avoid fatigue. They were given a snack and either a chance to enter a raffle or course credits for their participation.

### Fixations, narratives, and data quality

**Figure 5.**
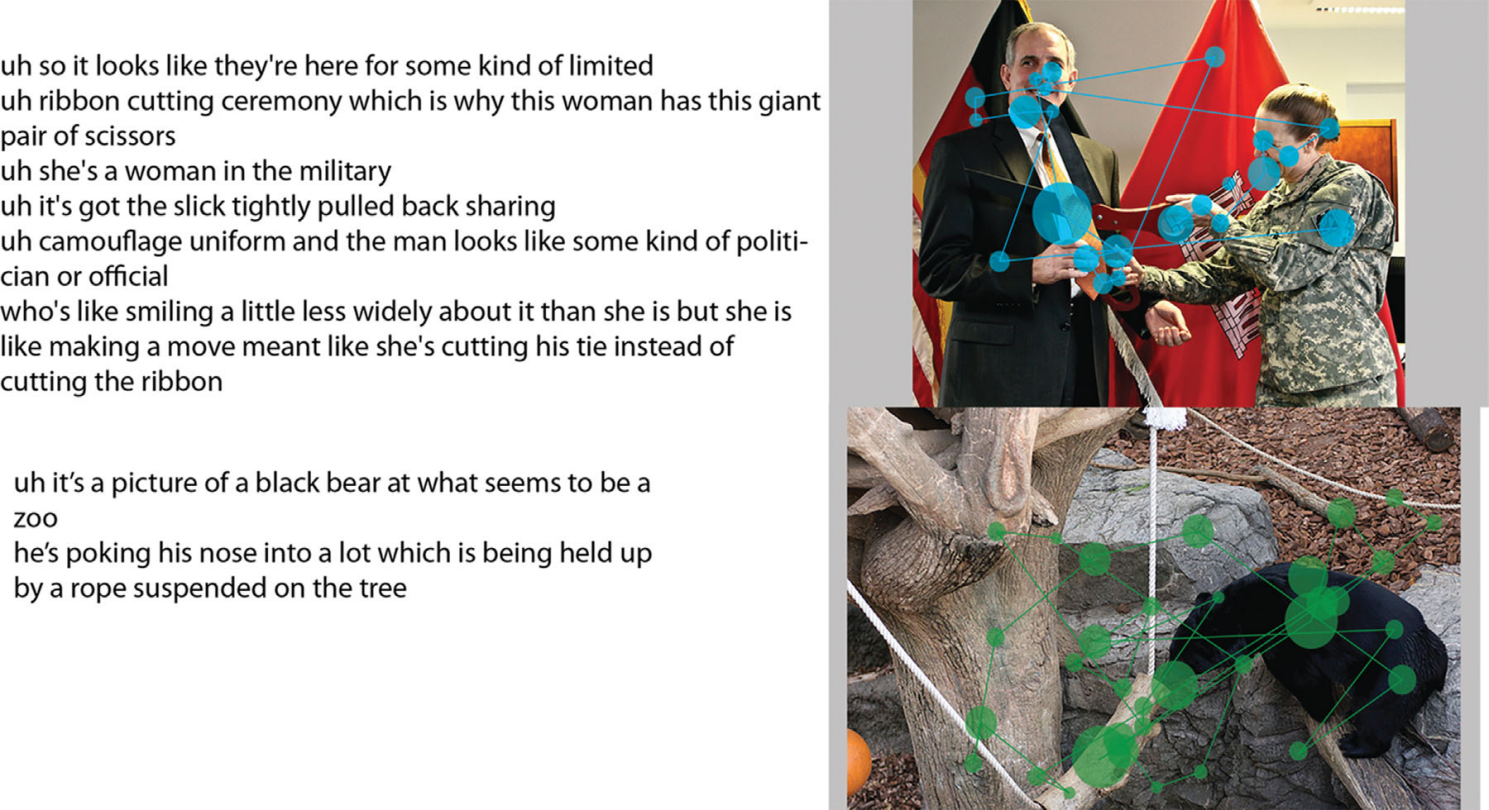
Co-captured multimodal data example. *Left:* Automated transcription of a participant's spoken description for two different images. *Right:* The eye movement data for the same participant overlaid on the corresponding images. The blue/green circles show fixations with the radius of the circles representing the duration of fixation. Saccades, connecting two fixations, are represented using the blue/green lines. Image credits: “Creative Commons Asian black bear” by Taro Sako, used under CC BY-NC 2.0. Scanpath overlaid on original, “USACE division visit to Europe District coincides with German Fasching celebrations” by U.S. Army Corps of Engineers Europe District, used under CC BY 2.0. Scanpath overlaid on original.

To detect the eye-tracking events we used the SMI software package BeGaze 3.1.117 with default parameters and a velocity-based algorithm. An example of the detected fixations is shown in Table 1. Because the accuracy of an eye tracker in use rarely meets the ideal-case value stated by the manufacturer (Wang et al., 2012a), we measured the data quality of all observers’ gaze data. We averaged over each participant's calibration data across the full trial to obtain an average calibration accuracy in the horizontal and vertical directions. We then calculated the overall average and standard deviation across all participants in the two directions. Participants whose averages in both directions were within two standard deviations of the overall average in that direction were included in a further analysis. Nine participants had a mean calibration and validation accuracy of more than two standard deviations in at least one direction and one participant had partial data loss. These 10 participants were removed from further analysis. The mean calibration accuracy for the dataset is reported in Table 2. The corpus size is 3000 instances of image descriptions (100 images × 30 participants), with 13 female participants and 17 male participants. Figure 5 shows an example of the scanpath, that is, fixations (blue/green circles) and saccades (blue/green connecting lines) of an observer overlaid on the corresponding image. Information about saccades is not used in our work.

**Table 1.** Sample raw data as obtained from the SMI eye tracker showing from left to right: system timestamp, left eye horizontal and vertical fixation locations, right eye horizontal and vertical locations, left eye and right eye event, respectively.

Time	*L*_*x*_[px]	*L*_*y*_[px]	*R*_*x*_[px]	*R*_*y*_[px]	L Event	R Event
7456470899	550.0	406.07	550.0	406.07	Fixation	Fixation

**Table 2.** Mean calibration accuracy across all participants and images. Some participants had a mean calibration accuracy of more than two standard deviations from the overall calibration accuracy and were not included in further analysis.

X Mean	X SD	Y Mean	Y SD	Participants	Images
0.67	0.25	0.74	0.27	30 (75%)	100

**Figure 6.**
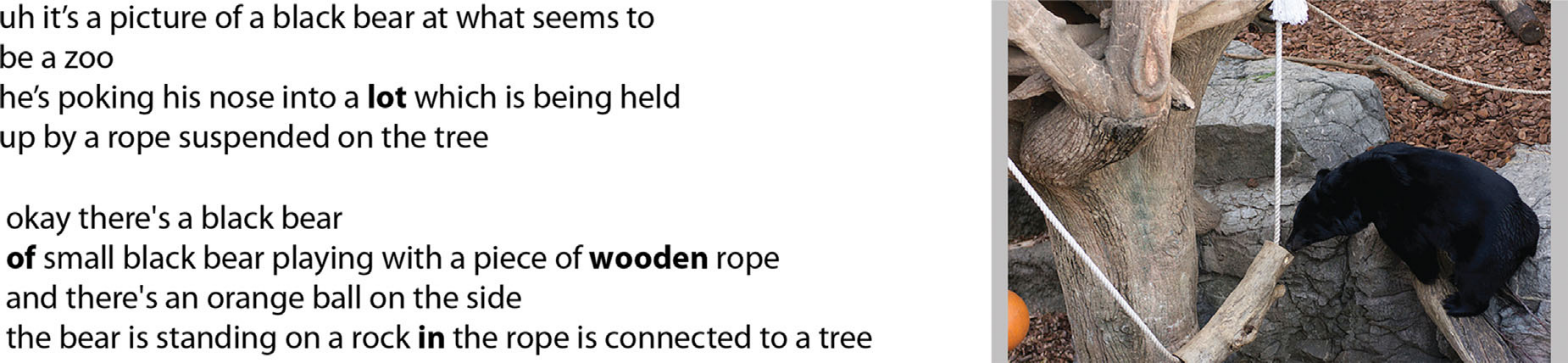
Examples of the transcribed speech for two participants obtained using IBM's Speech-to-Text ASR for the image shown at right. Whereas the narrative on the top left has only one incorrectly transcribed word (*lot* where the correct word is *log*), highlighting that using automated transcription can save manual labor, the narrative on the bottom left shows the limitations of ASR use with more word transcription errors. For the narrative on the bottom, the correct transcription for [*of, wooden, in*] are [*a, wood on, and*], respectively. Image credit: “Creative Commons Asian black bear” by Taro Sako, used under CC BY-NC 2.0.

We use the term *narrative* to refer to a participant's spoken description of an image, in which the speaker richly describes a depicted event in a visual environment. Our definition of narrative is characterized by description with a story-like progression, but is distinct from the narrow sense of narrative as a literary text. To fully automate data processing, the speech recordings of the narratives for the 30 participants for 100 images were machine-transcribed using the cloud-based IBM Watson Speech-to-Text service, an ASR system accessible via a Websocket connection^3^ (IBM, 2015). Example output is shown in Figure 5 (left). Figure 6 shows an additional comparison of output from the IBM Speech-to-Text tool for two observers. The transcription in Figure 6 (top left) contains few errors, which underscores the usefulness of using ASR rather than manual transcription of speech in large datasets. The ASR output shown in Figure 6 (bottom left), however, contains many errors, indicating that the use of ASR for transcription should be closely supervised. All of the spoken descriptions for a subset of five images from the snag dataset were manually corrected using (Praat Boersma, 2002) to be able to empirically explore the feasibility of substituting automatically generated transcriptions for careful but laborious manual transcriptions. The word error rate is 5%, which is state-of-the-art for ASR and is comparable with reported error rates of human transcription of conversational speech (Chiu et al., 2018). We discuss the comparison of annotation results using uncorrected and corrected transcripts for these five images later in the Results section.

### Gaze and narrative analysis

**Figure 7.**
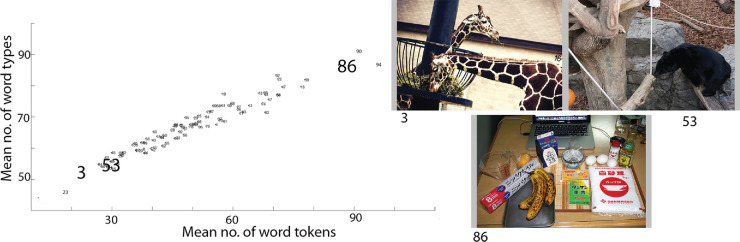
Scatter plot showing mean word types versuss. mean word tokens for each image across all observers. Each image is a data point. Highlighted images are shown at right. Image credit: “Giraffes” by Garret Voight, used under CC BY-NC 2.0, “Creative Commons Asian black bear” by Taro Sako, used under CC BY-NC 2.0, “I had three overripe bananas, and there is only one thing that can be made with overripe bananas” by Rachel+Micah, used under CC BY-NC-ND 2.0.

**Figure 8.**
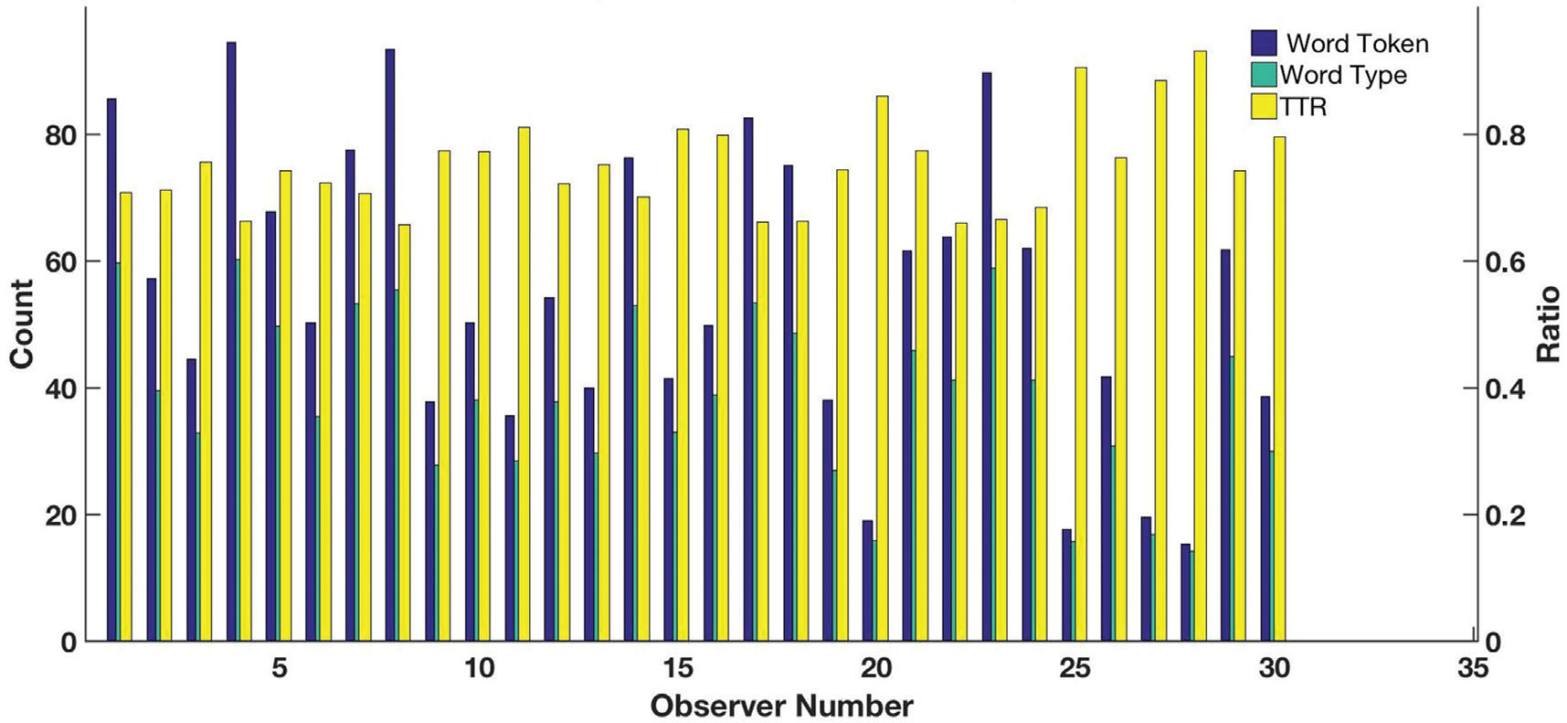
Bar plot showing the mean number of word tokens, word types, and type-token ratio (TTR) for each observer across the 100 images. All the observers have a mean type-token ratio of greater than 0.6, suggesting stronger lexical diversity. Observer number 28 has the highest mean type-token ratio.

Analyzing the gaze and narrative duration shows that the average fixation duration across the 30 participants was 250 milliseconds and average duration of narratives was about 22 seconds. We observed that on average observers viewed the image for approximately 0.58 seconds before uttering their description. Using the default NLTK word tokenizer (NLTK, 2015) we segmented the ASR-transcribed narratives into word tokens. Various measures for the first-order analysis of the narratives were then calculated. Table 3 shows the mean number of word tokens and word types, and mean type-token ratio across all the 3000 narratives (30 participants x 100 images) along with the standard deviation, minimum and maximum number of tokens, types, and type-token ratio. The mean number of tokens and the average duration of narratives together suggest that on average observers uttered 2.5 words per second. The mean type-token ratio of 75% in Table 3 suggests that there is significant lexical diversity across the dataset, supporting the richness of the dataset. Figure 7 shows a scatter plot for the mean number of word types against the mean number of word tokens for the 100 images. The plot is linear because a higher number of tokens typically result in higher number of types. Images 3 and 53 have fewer mean word tokens and types than image 86. For this dataset, this may be due to the number of significant objects in the images where a significant object is defined as an object that occupies a large area of the image. Images 3 and 53 have on average one or two objects, whereas image 86 has more than two. Comparing, the image number 3 has two significant objects (*two giraffes*) whereas image number 86 has more than five objects (*banana, eggs, foil, sugar, laptop*). The number of significant objects together with the task instruction may have resulted in the distribution obtained in Figure 7. We observe that a greater number of visually important regions in the image tend to result in a greater number of word tokens and types. Figure 8 shows the mean word tokens, mean word types, and mean type-token ratio for each observer across all the images. The high values of the mean type-token ratio suggest the lexical richness and heterogeneity present in the descriptions provided by the observers. In the following sections, we explore and discuss the applicability of the alignment-annotation framework to the snag dataset.

**Table 3.** Mean, standard deviation (SD), minimum, and maximum number of word tokens, word types, and type-token-ratio per narrative over 3,000 narratives (30 observers, 100 images). The high value of mean type-token ratio indicates greater lexical diversity.

	Mean	SD	Min.	Max.
No. of Tokens	55	31	5	295
No. of Types	38	17	5	132
Type-Token Ratio	0.75	0.11	0.41	1.00

## Alignment

**Figure 9.**
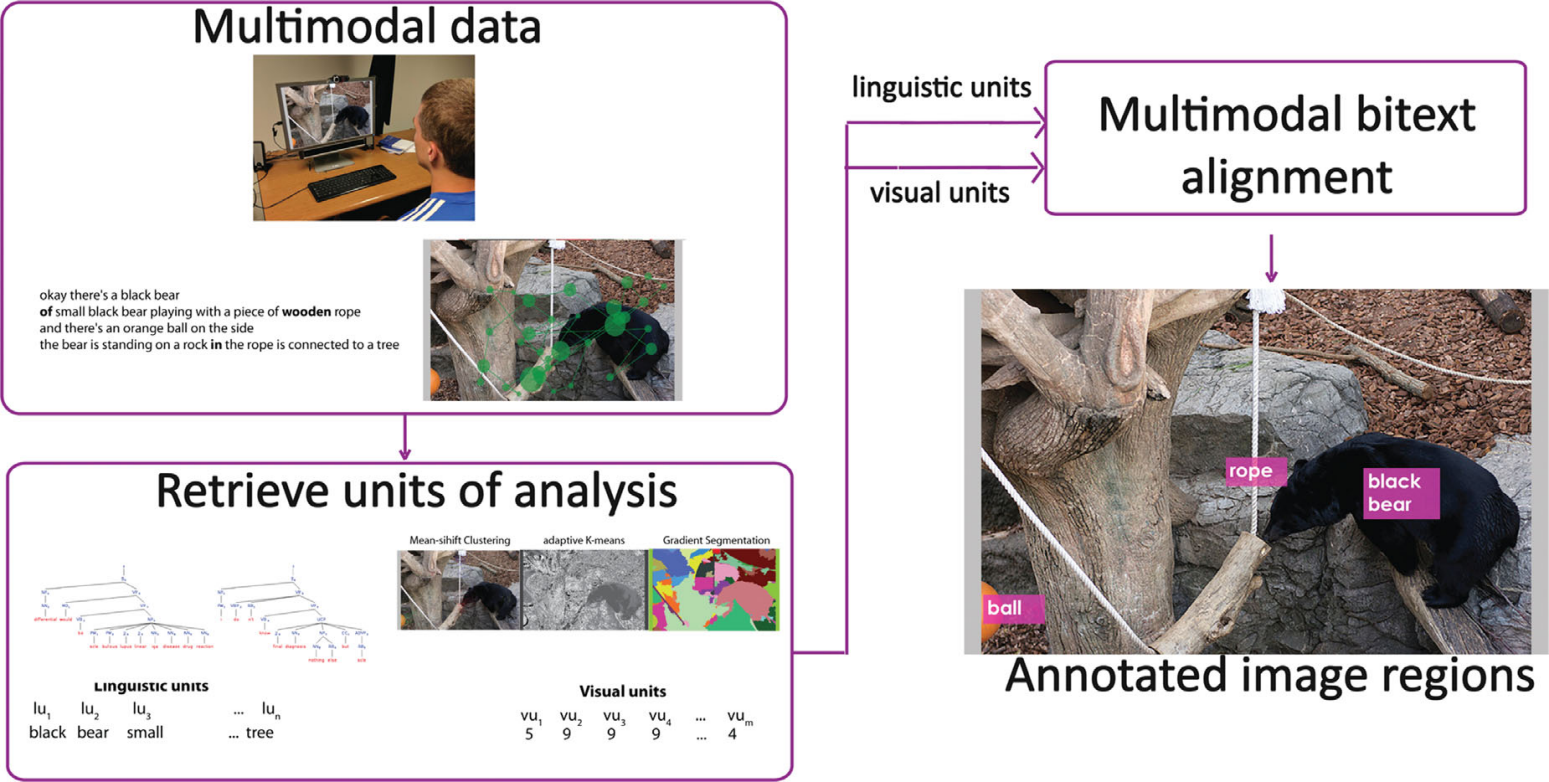
Implemented alignment-annotation framework. Step 1 involves collecting multimodal data, which is then processed to retrieve visual and linguistic units of analysis in step 2. In step 3, the units of analysis are fused using multimodal bitext alignment. In step 4, the alignment results are used to annotate image regions. Image credit: “Creative Commons Asian black bear” by Taro Sako, used under CC BY-NC 2.0. Scanpath and text overlaid on original. Also shown, “Segmented Bear”, is a derivative of “Creative Commons Asian black bear” by Taro Sako, used under CC BY-NC 2.0. “Segmented Bear” is licensed under CC BY-NC 2.0 by Preethi Vaidyanathan.

In this section, we describe the process to obtain linguistic units and visual units followed by the bitext alignment approach that the framework uses. We also discuss reference alignments we obtain from human annotators and two baseline alignments we use to compare against our framework. A flowchart showing the four main steps in our alignment annotation framework is shown in Figure 9.

### Linguistic units

**Figure 10.**
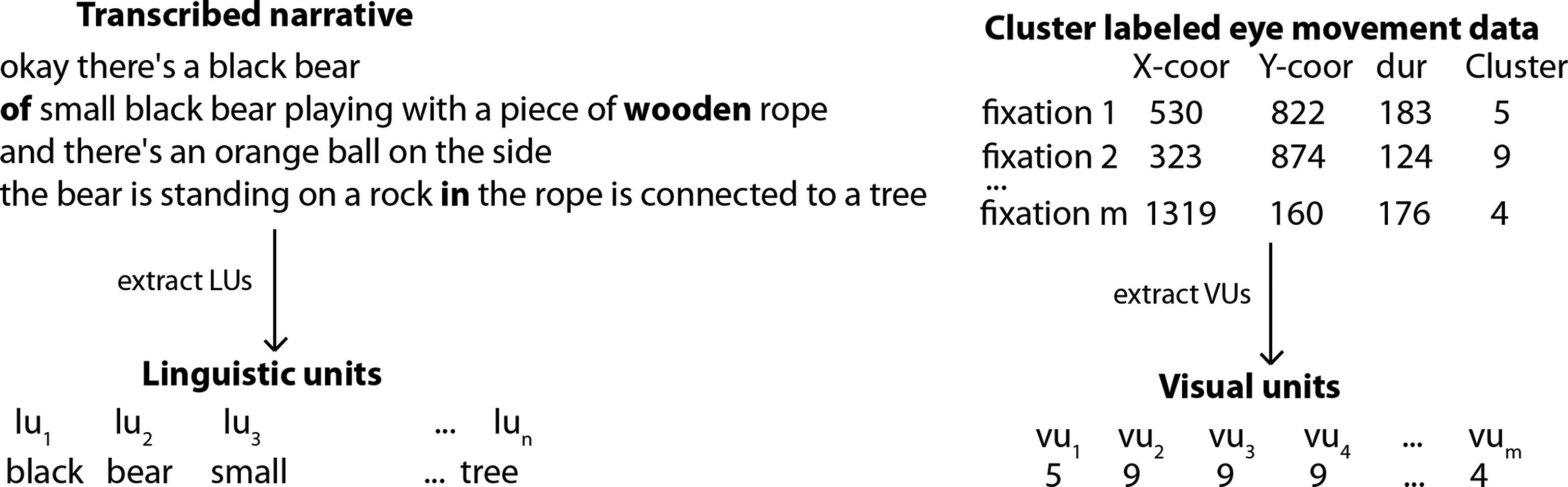
(*Left*) Process to extract linguistic units for an image. The original narrative is automatically transcribed using ASR and linguistic units are extracted. Transcription errors are not corrected manually to investigate their effect on the framework. Also, word tokens occurring only once per image are removed because they may not necessarily belong to any particular region in the image, occur owing to ASR errors or may reflect idiosyncratic word choices. *Right:* A similar process extracts visual units by labeling fixations based on the cluster they belong to according to the MSFC for a given image. In both cases, the linear order is maintained.

To automate the transcription process, we used the IBM Watson Speech-to-Text (ASR) service for automatic transcription of the audio recordings. Recordings of the descriptions were transmitted as .wav files over a WebSocket connection to the Speech-to-Text service which returned transcription results in JSON format. After performing minor text normalization, we parsed the transcripts with the Berkeley parser, using the English grammar that is included with this parser distribution (Petrov & Klein, 2007). From the parsed output we extracted all adjectives (e.g., *orange*), singular and plural nouns (e.g., *bear*), singular and plural proper nouns (e.g., *Achilles*), gerunds (e.g., *sniffing*), and foreign word tokens. The tokens were filtered to remove any remaining stopwords (e.g., *okay, some*) along with words used by the observers when following the task-specific instructions (e.g., *next*). Additionally, we removed any word tokens that were transcribed only once for a given image to avoid including ASR errors in our data. The frequency of word tokens in the narratives per image is a parameter that needs to be explored in depth. Importantly, throughout this preprocessing, the linear order of the linguistic units was maintained. Figure 10 shows an example of the linguistic units obtained for this dataset. There are some errors introduced by the ASR system such as *wood on* transcribed as *wooden*, which we do not correct. After comparing results with five images that were manually corrected (see subsection Effect of manual correction versus. ASR only), we chose to retain the automated transcriptions to investigate the result on the performance of the framework.

### Visual units

**Figure 11.**
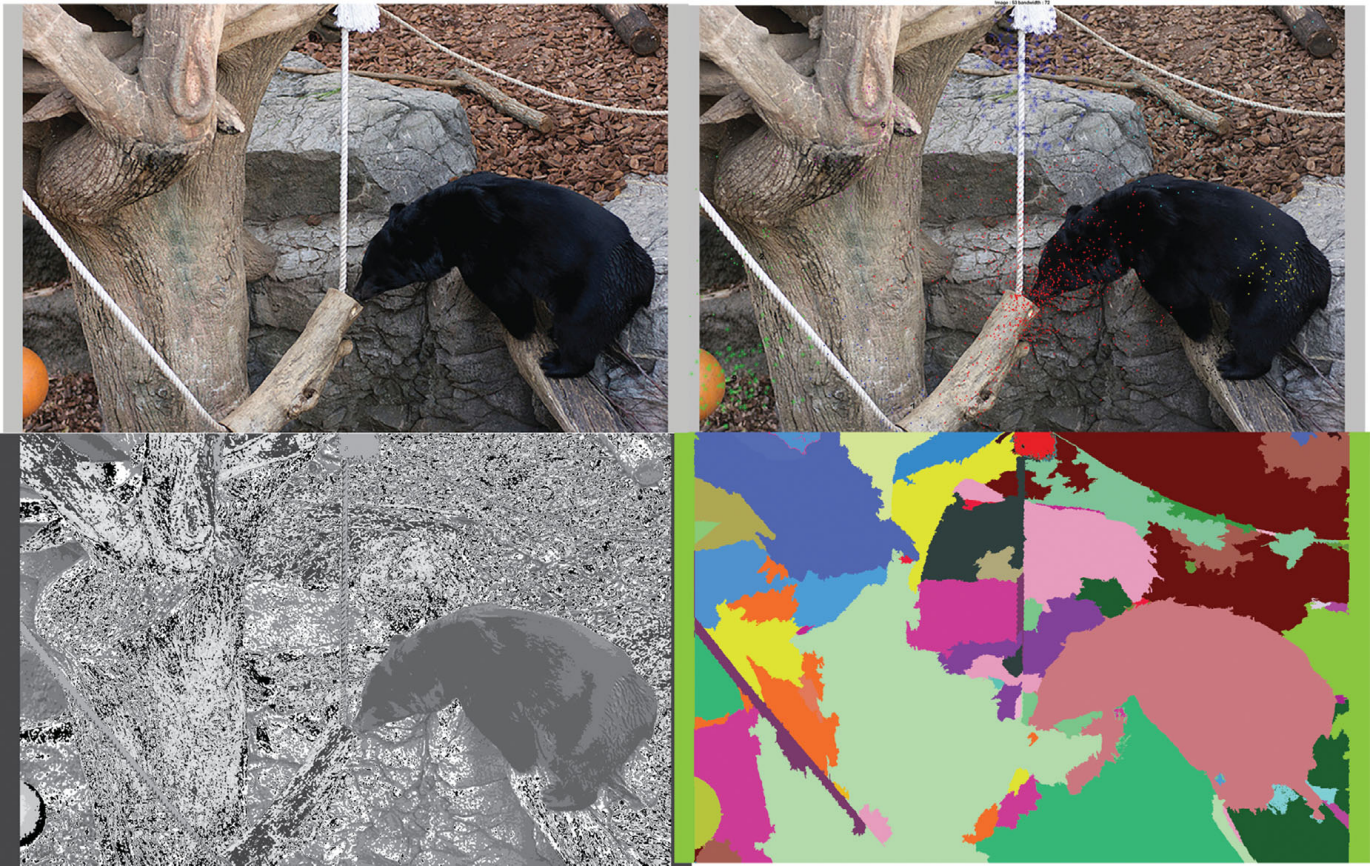
Original image (top left), MSFC (top right), *k*-means (bottom left, *k* = 8 for this image) and GSEG (bottom right) clustering or segmentation output for the image, used for extracting visual units. Image credit: This work, “Segmented Bear”, is a derivative of “Creative Commons Asian black bear” by Taro Sako, used under CC BY-NC 2.0. “Segmented Bear” is licensed under CC BY-NC 2.0 by Preethi Vaidyanathan.

Output from the eye tracker consisted of fixation locations given as (x, y) image coordinates and fixation durations per image per observer as shown in Figure 10. Visual inspection of the scanpaths of observers suggested existence of latent groups of fixations. To explore these emergent fixation clusters, we assigned fixations to image regions using three different techniques: mean shift fixation clustering (MSFC) (Santella & DeCarlo, 2004), Lloyd's *k*-means (Lloyd, 1982), and gradient segmentation (Ugarriza et al., 2009). The outputs of the three clustering or segmentation methods are shown in Figure 11.

The first technique was the MSFC algorithm. It is a data-driven method that clusters visual fixations into regions-of-interest. The advantage of MSFC over other techniques is that it does not require prior knowledge of the number of clusters and it is insensitive to outliers (Santella & DeCarlo, 2004). In this work we cluster the fixations spatially but also note that the same method could be used to cluster fixations temporally. MSFC was applied to each observer's eye-tracking data, assigning each fixation to a cluster in the image. Figure 11 shows fixations from all observers for one image. In this case, MSFC identified eight clusters. Clusters containing fixations outside of the image regions owing to blinks or track losses were discarded. For each observer, we then used this cluster information to obtain a linearly ordered sequence of *visual units* (i.e., image regions determined by fixations) that acted as the other input to the alignment algorithm, as shown in Figure 10. On average, MSFC yielded approximately 11 clusters per image. Fixations are encoded based on the cluster they belong to resulting in visual units. The linear order of the fixations is maintained.

In the *k-means* method, image pixels are divided into *k* clusters based on low-level image features. It is a fast, interpretable, and straightforward approach, but requires that the number *k* be determined *a priori*. We collected the *RGB* and spatial features for each image and applied Lloyd's *k*-means algorithm resulting in a segmented image. This is shown in the bottom left in Figure 11. For any given image, the number of clusters obtained using the MSFC is used as *k* for the *k*-means segmentation. The fixation sequence of each observer is overlaid on the segmented image and encoded using the segment label they fall within, without loss of linear order. The gradient segmentation (GSEG) method efficiently integrates spectral intensity, gradient, and texture information for segmentation purpose. It uses color space gradient information to identify clusters in an image, characterizes the texture in the identified clusters, and applies a region-merging procedure to generate a final segmentation. Sankaranarayanan Piramanayagam, a researcher at the Rochester Institute of Technology working on improving the GSEG algorithm, provided a toolbox that was applied to the snag images. Further mathematical details about GSEG can be found in Ugarriza et al. (2009). As with the other methods, fixations sequences are overlaid on the segmented image and encoded using the segment label.

### Bitext alignment

Studies have reported that fixations are generated before the end of words and that participants look at an object before naming it (Griffin & Bock, 2000). Our preliminary analysis showed that there is a temporal lag between when fixations on an object begin and when the person begins naming it (Vaidyanathan et al., 2012). For this reason, visual and linguistic units cannot be aligned merely by considering their time of occurrence. Instead, we require a method that can perform the alignment without making assumptions about the temporal relationship between the units. Conceptually, this is similar to translating one language into another in that the structural characteristics such as word order of the source language may not parallel those of the target language. We take advantage of this insight to explore whether a bitext alignment approach can discover meaningful alignments of multimodal data.

In statistical machine translation, bitext word alignment models are traditionally derived using a parallel corpus of sentences in which each sentence is rendered in two different languages. Table 4 shows a Hindi-English toy example. The principle behind bitext word alignment is as follows: proceed through each pair of training sentences, keeping track of the number of times words co-occur in the two languages. These counts are iteratively used to estimate the probability that a word in one language is a translation of (aligns with) each possible word in the other language. In the toy example above, a bitext alignment model would eventually estimate a high probability that the Hindi word *ghar* is a translation of (aligns with) the English word *house*. In the multimodal scenario of this study, the linguistic (nouns, adjectives, gerunds, and foreign words) and visual (numeric labels of cluster/segments) units extracted for an image represent a pair of “sentences” in the training data.

**Table 4.** Toy example illustrating the bitext alignment between Hindi and English sentences. The probability of English word *house* being a translation of Hindi word *ghar* in the first pair of sentences is small. Over time this probability increases (black to red to blue) as more parallel sentences containing the two words are added to the training data. Similarly, the bitext alignment algorithm keeps track of the number of times the Hindi word *chhota* occurs in parallel to the English word *small*.

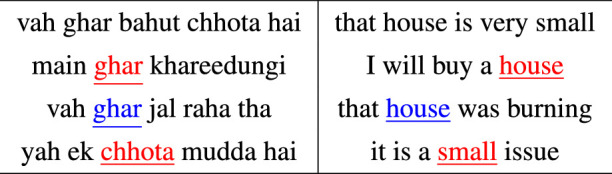	

**Figure 12.**
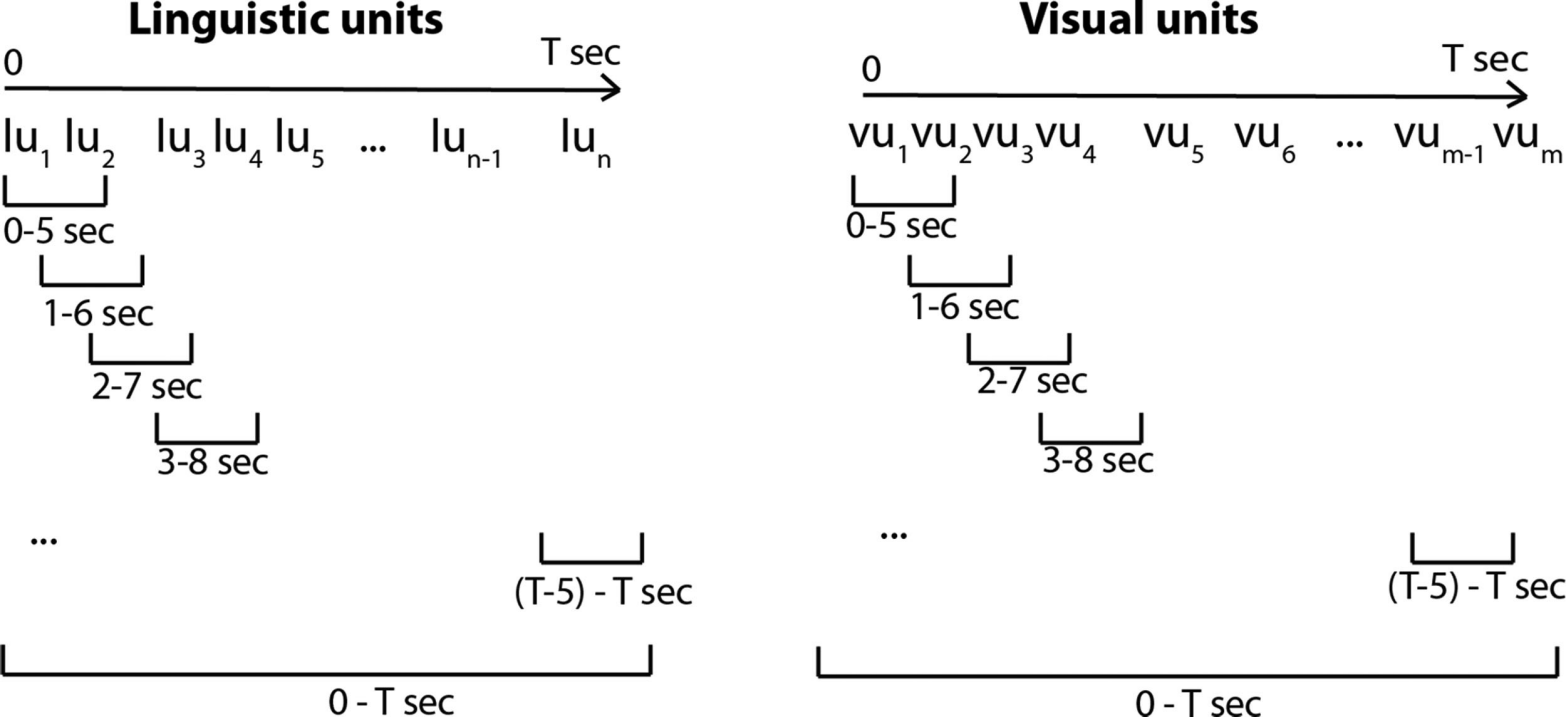
(*Left*) Linearly ordered linguistic units obtained from the transcribed narrative. (*Right*) Linearly ordered visual units obtained by labeling fixations using the MSFC algorithm. The labels are different when using other segmentation methods for identifying visual units. Note the linguistic units or visual units are not isochronous. Therefore, the number of linguistic units or visual units between the sliding windows may be different.

Using a sliding window of *T* seconds, linguistic and visual units within each sliding window are extracted and added as additional “sentences” or multimodal data pairs to the corpus, as shown in Figure 12. Therefore, the number of linguistic or visual units can be different between the sliding windows. By applying the sliding window, the parallel corpus grows substantially. The original linguistic and visual unit sequence pair, on which the sliding window is applied, is also included in the training data. We use the sliding window for two reasons. First, the narratives are long, which is very challenging for expectation maximization-based word alignment. A windowing approach allows us to break each narrative into smaller chunks. In addition, windowing allows us to expand the number of parallel sentences for each image, from only 30 to several hundred or more parallel sentences, which results in a more robust model. Second, as we have noted, people do not say words at the exact time they look at regions corresponding to those words. A sliding overlapping window has the potential to capture this visual-linguistic behavior.

**Figure 13.**
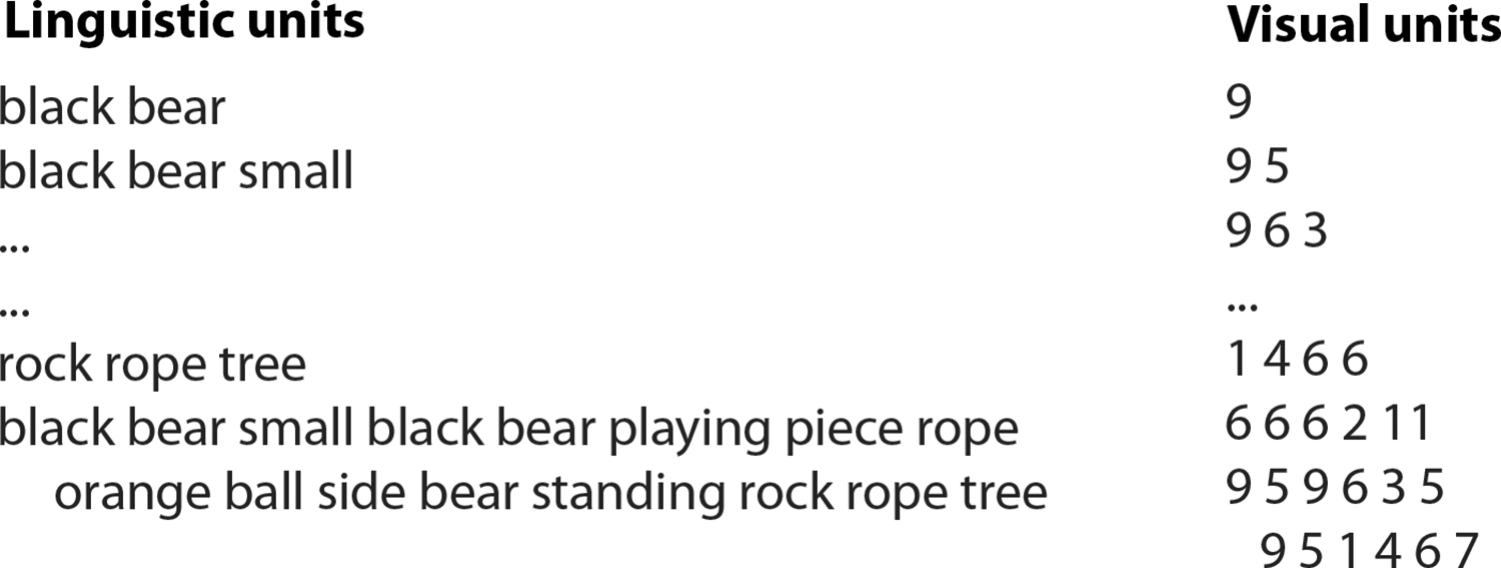
Example training data: A sliding window of 5 seconds is applied to the pair of visual and linguistic “sentences” to expand the data. Subsequently, contiguous visual units are merged and visual units with longest fixation duration are selected. The selected visual units, together with the linguistic units, comprise the training data.

An example of our training data is shown in Figure 13.

Another complication in using this multimodal data is that the sequences of visual units are substantially longer than the sequences of linguistic units. In order to balance the sequence lengths, we merge contiguous identical visual units (e.g., *cluster3*, *cluster2*, *cluster2*, *cluster3* is converted to *cluster3*, *cluster2*, *cluster3*). This is applied to each sliding window. Subsequently, visual units with the longest fixation duration are selected (keeping the linear order intact) based on the visual-linguistic ratio. The visual-linguistic ratio is defined as $\beta =\frac{Numberofvisualunits}{Numberoflinguisticunits}$, where β = 1 results in an equal number of visual and linguistic units within each data pair. We also report on the impact of changing the value of *T* and β as well as the visual unit selection method (α), on the framework's performance.^4^. Using this method, the training data for each image increased to approximately 1000 sentences.

We use the Berkeley aligner (Liang et al., 2006) rather than Giza++ (Och et al., 2000) because of its reported greater alignment accuracy and flexibility in testing an existing alignment model on unseen data. One of the greatest strengths of the Berkeley aligner is the use of joint training. Further details can be found in Liang et al. (2006). The Berkeley aligner was run with default parameters settings (two iterations each of IBM Model 1 and an HMM, joint training, and posterior decoding) with the exception of the posterior threshold used for decoding, which was lowered to 0.1. This value was empirically determined to maximize alignment accuracy on a small held-out set of multimodal data.

### Reference alignments

**Figure 14.**
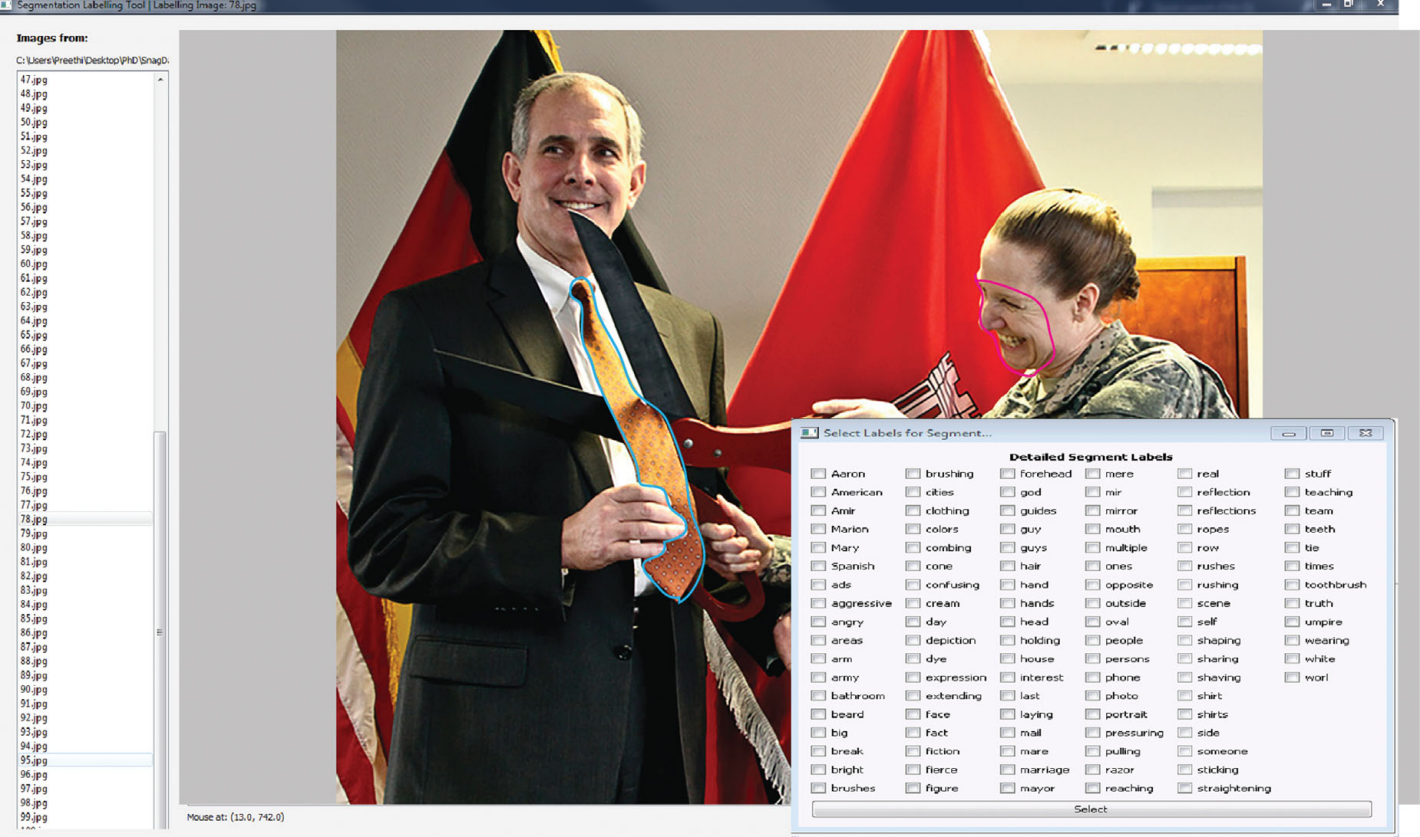
RegionLabeler GUI used to acquire reference alignments. The person preparing the manual alignments is able to draw borders with a mouse around regions and label them with linguistic units. For this image, all pixels within the blue border are marked as *tie* in the sure alignments whereas all pixels within the pink border are marked as *laughing* in the possible alignments. Image credit: “USACE division visit to Europe District coincides with German Fasching celebrations” by U.S. Army Corps of Engineers Europe District, used under CC BY 2.0. Region outlines overlaid on original.

Reference alignments (ground truth) were prepared using a GUI called RegionLabeler^5^ (Vaidyanathan et al., 2018) to allow evaluation of the resulting multimodal alignments. This represented the manual alignments obtained by associating each fixation cluster in the case of MFSC and image segment in the case of image segmentation with its corresponding word tokens (linguistic units). Figure 14 shows a screenshot of the GUI developed specifically to allow the annotator to perform the manual alignments by drawing borders around image regions and then selecting linguistic units from a pop-up box that contains all the linguistic units for that image. The output from the GUI consists of sets of image pixel coordinates labeled with one or more associated linguistic units, which are then processed to obtain linguistic units corresponding to either fixation clusters in the case of MSFC or image segments in the case of *k*-means and GSEG. The annotator specifies two kinds of alignments: sure (S) and possible (P) (Och & Ney, 2003). sure alignments define alignments where there is no ambiguity. For example, for the image in Figure 14, the annotator aligned the word *tie* to the image region marked in blue. This alignment is therefore added to the set of sure reference alignments (set S). In cases where there was ambiguity in whether a word represented the marked region, the word was added to the possible alignments (set P). Och and Ney (2003) use possible alignments to accommodate idiomatic expressions, free translations, and missing functions words, for example, when a preposition in one language does not have a direct translation in the other language, possible alignments allow the aligner not to be penalized for not aligning the preposition to the verb or the article. Slightly differently, we use possible alignments to capture words that are ambiguous in whether they correspond to a region. For example, for the image in Figure 14, the annotator was not absolutely certain if the word *laughing* belongs to the image region marked in pink, thereby adding this alignment pair to the possible reference alignment (set P). The amount of overlap between narratives and reference data is shown in Table 5.

**Table 5.** Percent linguistic units present in both the narratives and the images for the general-domain snag dataset.

Total no. of linguistic units in narratives	34621
No. of linguistic units in narratives and images	25225
% of linguistic units in narratives and images	72.86

Each concrete noun or adjective that was used by an observer was presented to the annotator, and the annotator simply had to indicate which regions in the image corresponded to that word. Most speakers in a speech community share terminology to refer to objects. For these reasons, we used only one annotator and given the general-domain nature of this dataset, the first author of this work performed both the sure and possible manual alignments. All the manual alignments were done using the post-filtered word tokens. We observed that not all the linguistic units present in the narratives were present in the image. Therefore, these linguistic units would also be absent from the reference alignments that are used for evaluation. The percent of linguistic units present in the narratives that are also present in the image is close to three-fourths.

### Baseline alignments

**Figure 15.**
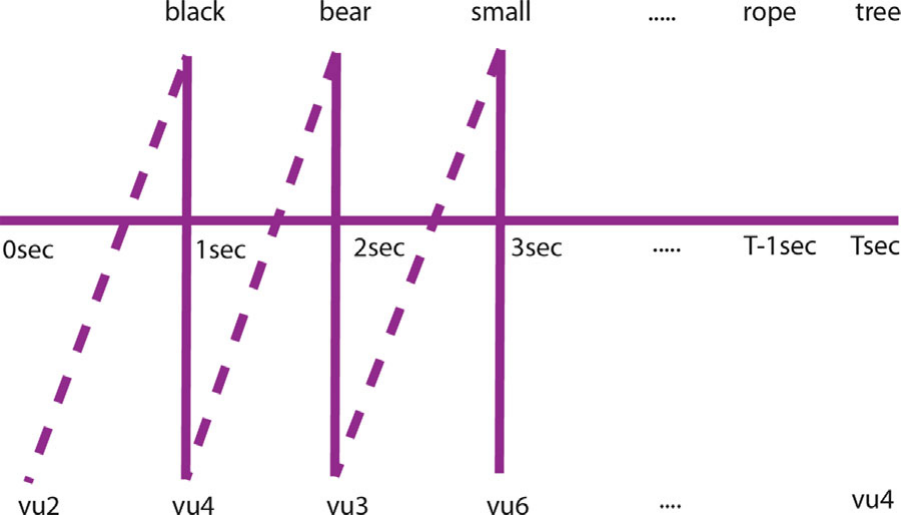
Visual units (bottom) are aligned with linguistic units (top) uttered simultaneously (solid line) and after a 1-second delay (dashed line) for the image shown in Figure 11.

We compare the performance of the proposed alignment method with two temporal methods of alignment, namely *simultaneous* and *1-second delay* baselines. Figure 15 shows the simultaneous (solid line) and 1-second delay (dashed line) baseline for an example set of visual and linguistic units. Simultaneous baseline alignments are obtained assuming that the observers utter the word corresponding to a region at the exact moment their eyes fixate on that region. The 1-second delay baseline assumes that there is a 1-second delay between a fixation and the utterance of the region label, based on prior research (Griffin, 2004). Although the amount of delay is a parameter that can be varied for comparison against the proposed alignment, it is unlikely that a fixed-delay alignment will be sufficient. Prior research has shown that the delay between when a person looks at an object and mentions it depends on factors such as usage frequency and complexity of the object's name (Griffin & Bock, 2000) and complexity of the image (Vaidyanathan et al., 2012).

## Results and discussion

### Evaluation of results

**Figure 16.**
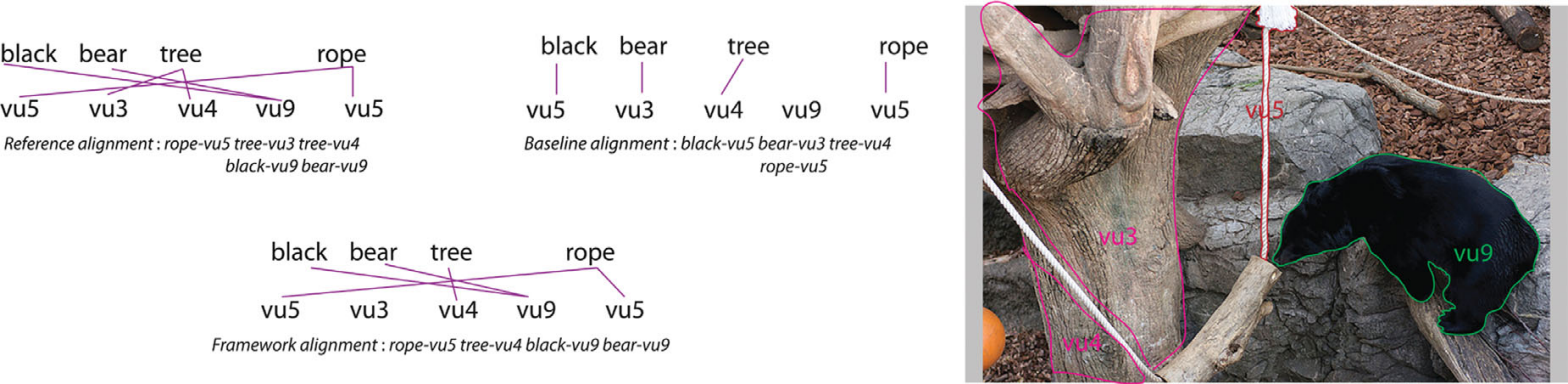
Example illustrating output from our framework, the reference alignment, and baseline alignment for a given pair of linguistic and visual “sentences.” Image credit: “Creative Commons Asian black bear” by Taro Sako, used under CC BY-NC 2.0. Region labels and boundaries overlaid on original.

Figure 16 shows the framework output for a given linguistic and visual “sentence” pair. We use the following metrics and equations from Och and Ney (2003) to test how well the framework identifies the correct word-region correspondences compared with the reference alignments:

$$Precision=\frac{\left|A\cap P\right|}{\left|A\right|}$$

$$Recall=\frac{\left|A\cap S\right|}{\left|S\right|}$$

$$Alignment\;error\;rate=1-\frac{\left|A\cap S|+|A\cap P\right|}{\left|A|+|S\right|}$$

where A is the set of alignment pairs in the output alignment, S is the set of sure alignments in the reference, and P is the set of possible alignments in the reference. AER is the alignment error rate, which is commonly used to evaluate word alignment in machine translation. A high precision and recall resulting in a low AER is considered good. The image regions and their labels change with the segmentation technique being used. Therefore, each segmentation method has its own set of simultaneous and 1-second delay baselines, reference alignments, and alignments from the proposed framework that are used to compute the metrics. In general, the 1-second delay baseline tends to perform as well as or better than the simultaneous match baseline.

**Figure 17.**
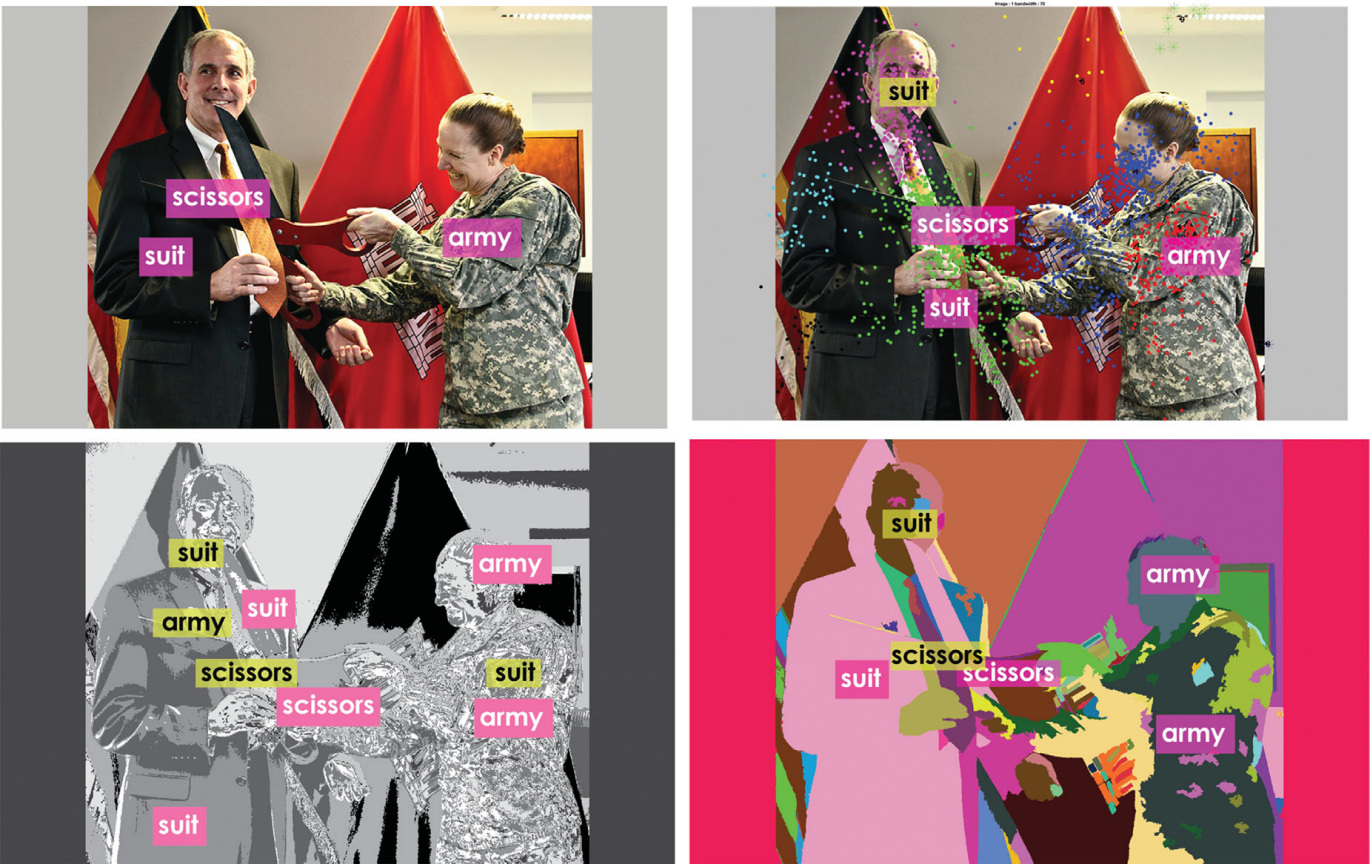
(*Top left*) Reference alignments as provided by the annotator. Alignment output when using the (*top right*) MSFC, (*bottom left*) *k*-means, and (*bottom right*) GSEG methods, respectively. Correct alignments are shown in *pink*, whereas misalignments are shown in yellow. MSFC has fewer misalignments compared with *k*-means and GSEG methods. The visualization tool places the label within the corresponding segment, however, in cases where the segments are small, the labels may seem to belong to the adjacent segments too (e.g., *scissors* in *bottom right*). Image credit: This work, “Annotated tie-cutting,” is a derivative of “USACE division visit to Europe District coincides with German Fasching celebrations” by U.S. Army Corps of Engineers Europe District, used under CC BY 2.0. “Annotated tie-cutting” is licensed under CC BY 2.0 by Preethi Vaidyanathan.

**Figure 18.**
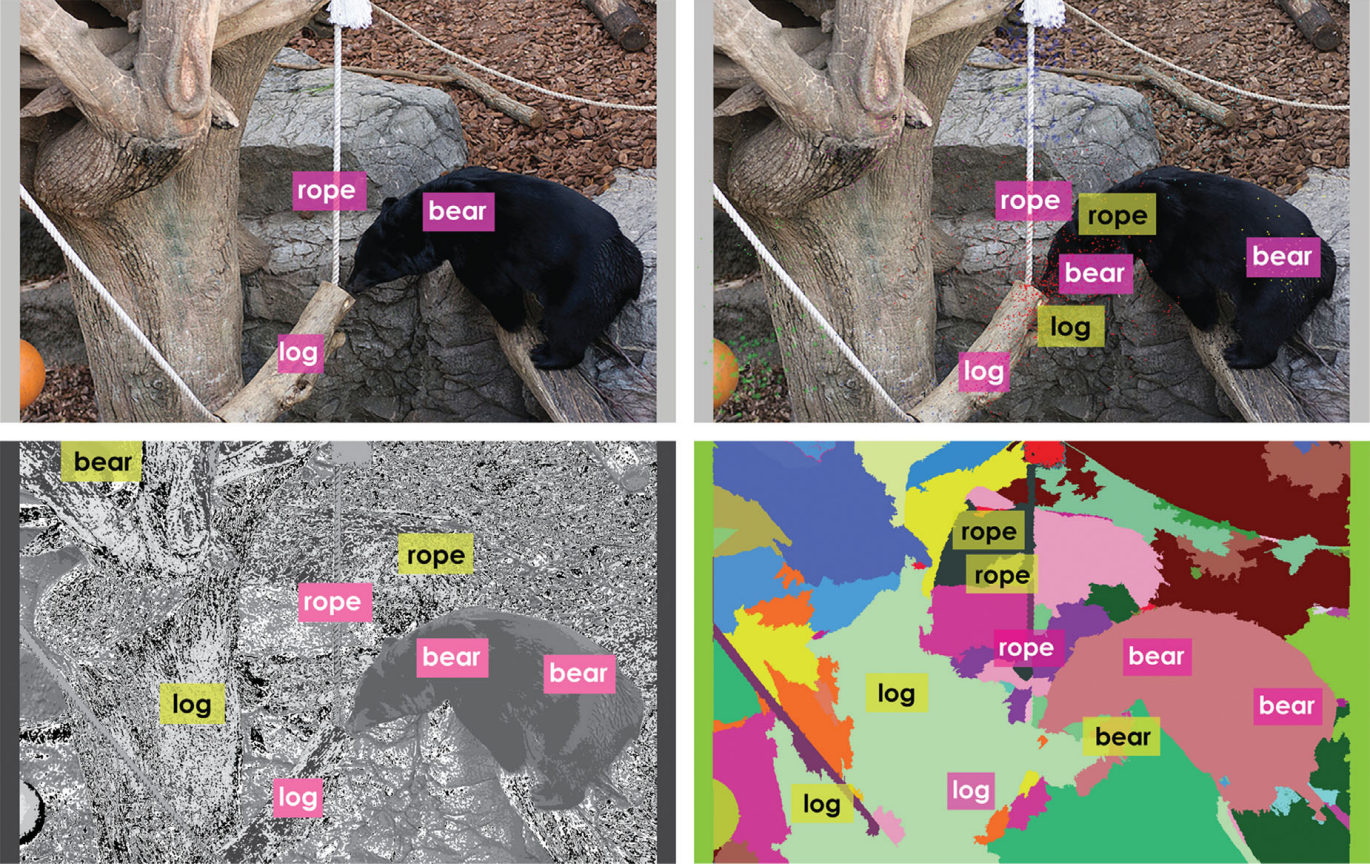
Annotation results for a different image with reference alignments in the top left, MSFC in the *top right*, *k*-means in the *bottom left*, and GSEG in the *bottom right*. Again, correct alignments are shown in *pink*, whereas misalignments as well as labels not belonging to reference alignments are shown in yellow. Note that, as in the previous case, both *k*-means and GSEG tend to misalign more often than MSFC. Image credit: This work, “Annotated Bear,” is a derivative of “Creative Commons Asian black bear” by Taro Sako, used under CC BY-NC 2.0. “Annotated Bear” is licensed under CC BY-NC 2.0 by Preethi Vaidyanathan.

Alignment Plotter,^6^ a qualitative visualizer, was built to visualize the resulting annotations corresponding to the image regions. The visualizer sorts the words in increasing order of frequency of utterance and displays *W* words on the corresponding image region locations. The number of visualized words *W*, if needed, can be different for different images. Various results shown and discussed in this article use the visualizer with the value of *W* ranging from 2 to 4 (e.g., see Figures 17 and 18) in order to illustrate the output annotations. Low values of *W* were picked to avoid clutter for illustration of results.

### Alignment annotation results

We calculated the average precision, recall, and AER and compared them against the baselines. The comparison was done for three clustering or segmentation methods: MSFC, *k*-means with *RGB* color features, and *k* equal to the number of fixation clusters obtained by MSFC for each image, and GSEG.

The simultaneous baseline's performance measures are similar to the 1-second delay baseline. As shown in Table 6, the proposed framework for alignment performs better than either of the baselines. Among the three clustering or segmentation methods, MSFC yields the highest recall and lowest AER. It achieves an absolute improvement of 0%, 19%, and 10% for precision, recall, and AER, respectively, over the 1-second delay baseline. The absolute improvement percentages are shown in the last row of Table 6. In contrast, *k*-means with k equal to the number of clusters from MSFC results in greater precision with an absolute improvement of 6%, 14%, and 14% over the 1-second delay baseline for precision, recall, and AER, respectively. In comparison with MSFC and *k*-means, the performance of GSEG is comparable with an absolute improvement of 6%, 13%, and 13% for precision, recall, and AER, respectively. Table 7 shows the performance for each clustering or segmentation method based on the number of images. Although all three methods yield higher recall and lower AER than baseline for almost all 100 images, *k*-means and GSEG yield higher improvement in precision for 96 images outperforming MSFC.

**Table 6.** Average alignment performance across images for three different clustering or segmentation methods. Our framework with the MSFC clustering method provides the best recall and lowest AER (as indicated in bold). However, *k*-means provides the best precision (highlighted in bold). The absolute improvement achieved by the different clustering or segmentation methods over the 1-second delay baseline are shown in the last row.

	MSFC			*k*-means			GSEG		
	Precision	Recall	AER	Precision	Recall	AER	Precision	Recall	AER
Simultaneous	0.42	0.30	0.65	0.49	0.17	0.74	0.41	0.14	0.78
1-second delay	0.43	0.31	0.64	0.50	0.17	0.74	0.42	0.15	0.78
Alignment framework	0.43	**0.50**	**0.54**	**0.56**	0.31	0.60	0.48	0.28	0.65
% improvement (over 1-second delay)	0	19	10	6	14	14	6	13	13

**Table 7.** Number of images for which our alignment framework provides an improvement over the baselines, for each case of clustering or segmentation method. All three methods provide improvement over the baselines for both recall and AER on all images with *k*-means and GSEG providing improvement in precision as well. The total number of images used in the dataset was 100.

	MSFC	*k*-means	GSEG
Precision	62	96	96
Recall	100	100	100
AER	99	100	100

A visual comparison of reference alignments provided by the annotator with the alignments obtained through our framework for the three clustering or segmentation methods shows (Figures 17, 18) most of the words are correctly aligned (pink) by all three methods. MSFC correctly aligns many labels present in the sure reference alignments such as *army, scissors*, yielding a higher recall. It also aligns some of these labels such as *suit* to regions they do not belong to explaining the low precision values. Both *k*-means and GSEG misalign labels such as *suit, scissors* more often leading to a lower precision in comparison with MSFC. Annotation results for a different image are shown in Figure 18. Again, MSFC seems to correctly align labels more often than *k*-means and GSEG methods.

Many state-of-the-art image annotation methods involve humans superficially, for example, for marking objects in the images (Karpathy & Fei-Fei, 2015; Anderson et al., 2018). Some recent work involves humans at a deeper level, but they limit the observer's vocabulary (Gygli & Ferrari, 2018) or lack the benefit of multimodal information (Vasudevan et al., 2018b). Some computer vision and deep learning approaches that involve multimodal information such as gaze and speech are constrained to objects in the image, as well as objects learned through annotation (Vasudevan et al., 2018a). In contrast, our framework provides the affordances of the human-centered gaze and language-based approach. For instance, for some images where the subject in the image is directly looking at the camera that was used to take the picture, our framework annotates the subject with the term *camera*, although the camera itself is not in the picture. Our framework can capture the human interpretation of this perspective and the narrative description allows people to take different approaches to interpreting what is noticeable in an image. However, such abstract labels that are not present in either sure or possible reference alignments decrease the precision values of the framework. The improvement over the baselines suggests that purely temporal alignment of fixations and utterances is insufficient for region annotation and underscores the promise of our alternative alignment-annotation approach. This is true regardless of the method used for the identification of visual units or the type of image. These results indicate that the alignment-annotation framework could in the future consist of a clustering or segmentation method that uses both fixations and image features during the segmentation process. This will help to decrease the likelihood of image regions representing different concept labels correspond to the same region label.

**Figure 19.**
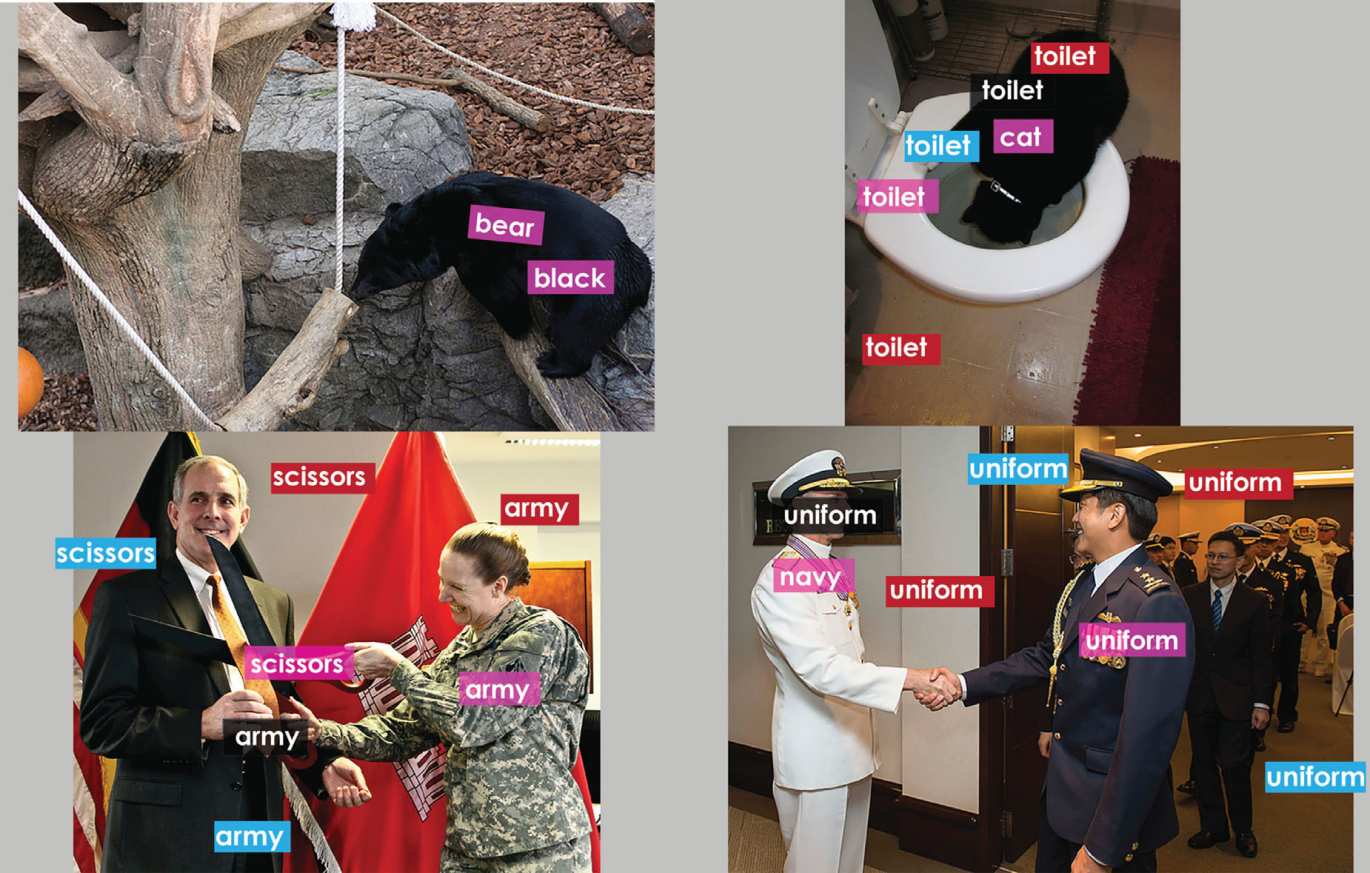
Example images from category top left: O = 1, with one primary object (*bear*). Top right: O = 2, two primary objects (*cat, toilet*). Bottom left: O = 3, three primary objects (*gentleman, army officer, scissors*). Bottom right: O≥4, four or more primary objects (*person 1, person 2, person 3, person 4, etc*), respectively. Labels in *pink* indicate all the three methods correctly aligned them. Incorrect alignments are shown in *black* (MSFC), *red* (*k*-means), and *blue* (GSEG). The number of misalignments increases as the images get more cluttered. Image credit clockwise: “Creative Commons Asian black bear” by Taro Sako, used under CC BY-NC 2.0. Region labels and boundaries overlaid on original. “Freshest Water in the House” by Megan, used under CC BY-NC 2.0. Region labels and boundaries overlaid on original. USACE division visit to Europe District coincides with German Fasching celebrations by U.S. Army Corps of Engineers Europe District, used under CC BY 2.0. Region labels and boundaries overlaid on original. “130513-N-WL435-087” by U.S. Pacific Fleet, used under CC BY-NC 2.0. Region labels and boundaries overlaid on original.

To study the impact of image complexity on the annotations, we divided the images in the snag dataset into four categories ranging from simple to complex, as shown in Figure 19. Category *O = 1* consisted of images with one primary object to gaze at and describe. For instance, image on the top left of Figure 19 consists of one prominent object *bear*. Although there are other objects in the image to look at and describe since there is only one prominent object the annotator categorized this image in *O = 1* category. Likewise, category *O = 2* and *O = 3* consisted of two and three primary objects to gaze at and describe. Category *O ≥ 4* represents images with more than three primary objects. There were 16, 37, 12, and 35 images in each category, respectively. The MSFC yielded on average 11, 10, 11, and 11 clusters for the four categories, respectively. The *k*-means resulted in the same number of segments for each category because it uses the number of clusters provided by MSFC. As indicated in Table 8, the categorization does not have much of an effect on the general trend of performance of the clustering or segmentation methods. MSFC claims high recall and low AER values while *k*-means claims high precision. However, the best performance is obtained for images in category *O = 2* followed by category *O = 1*. This finding that the number of objects in an image may affect the alignment framework's performance. This categorization is coarse and may involve subjectivity because it was performed by one annotator, the primary researcher. Further work is required to explore dividing the images based on number of objects and using more annotators to reduce subjectivity.

**Table 8.** Comparison of alignment performance for four different categories of images for different clustering or segmentation methods. These four categories are defined based on the approximate number of primary objects in the image, for example O = 1 indicates the images in this category had one primary object to gaze at and describe. Not surprisingly, as the number of primary objects increase, the alignment performance decreases. Also, regardless of the category of image, *k*-means provides the best precision whereas MSFC provides best recall and AER (as indicated in bold).

	Precision			Recall			AER		
	MSFC	*k*-means	GSEG	MSFC	*k*-means	GSEG	MSFC	*k*-means	GSEG
O = 1 (16)	0.43	0.57	0.47	**0.55**	0.31	0.3	0.53	0.59	0.63
O = 2 (37)	0.47	**0.59**	0.51	**0.55**	0.32	0.29	**0.51**	0.58	0.63
O = 3 (12)	0.44	0.55	0.48	0.44	0.28	0.25	0.56	0.62	0.67
O ≥ 4 (35)	0.38	0.51	0.44	0.47	0.29	0.27	0.59	0.63	0.66

Figure 19 shows the obtained alignments overlaid on their respective images for the four categories. In general, labels are aligned correctly, but we also get some misalignments, regardless of the clustering or segmentation method used. These misalignments seem to increase in number as the complexity of an image (i.e., number of objects) increases, thereby lowering performance. MSFC seems to have fewer spurious alignments compared with *k*-means and GSEG, possibly because it is derived entirely from the fixation data. An increase in the number of primary visual units (i.e., objects to look at) stimulates lexical diversity in the description. Another factor is object occlusion resulting in objects being less distinguishable and separated. This reduced frequency of fixated regions (visual units) or uttered words (linguistic units) impacts the performance; in particular, the visual units seem to be more prone to misselection because of the variations in eye movements among observers. Using part-whole relationships for both the visual and linguistic data would help to address these issues.

**Figure 20.**
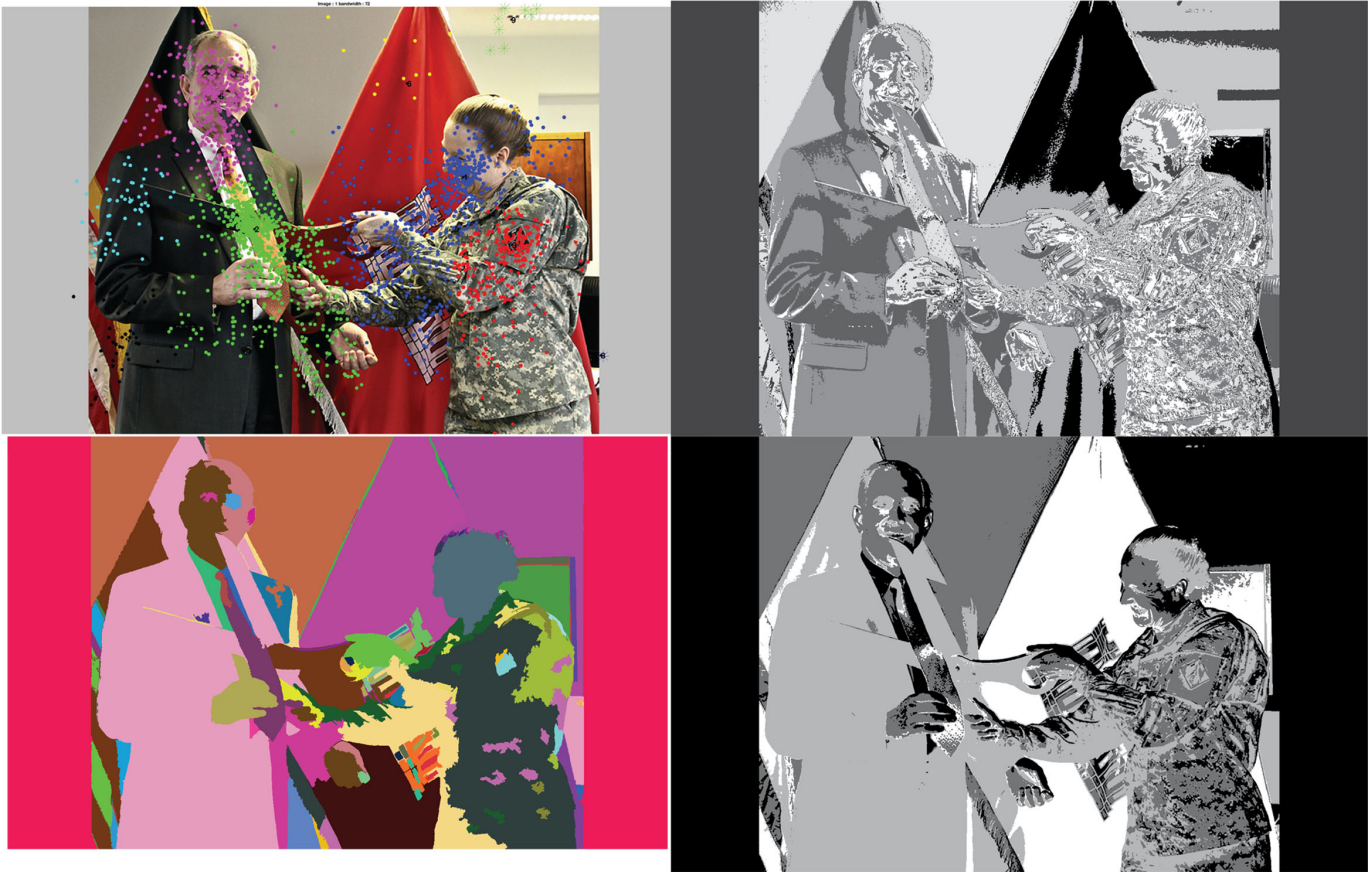
Output from top left: MSFC, *top right*: *k*-means, where *k* is equal to the number of clusters obtained from MSFC, *bottom left*: GSEG, and *bottom right*: *k*-means, where *k* = 4, respectively. The *k*-means and GSEG tend to oversegment leading to multiple segment labels for a given word-label whereas *k* = 4 may lead to undersegmentation in other cases leading to one segment-label shared by various word-labels. A semantic segmentation method built using gaze data and image features may be the solution to this issue. Image credit: This work, “Segmented tie cutting”, is a derivative of “USACE division visit to Europe District coincides with German Fasching celebrations” by U.S. Army Corps of Engineers Europe District, used under CC BY 2.0. “Segmented tie-cutting” is licensed under CC BY 2.0 by Preethi Vaidyanathan.

As previously mentioned, MSFC is less sensitive to the errors introduced owing to sharing of image features by various objects. Sharing of image features can lead to common image segment-labels during the segmentation process. For example, in both GSEG and *k*-means (Figure 20), the man's *coat* and part of the *scissors* have the same segment-label. This would lead the framework to incorrectly learn that labels *coat* and *scissors* both belong to the same image region, increasing the AER. For our purposes, the image region corresponding with the word *scissors* need not be segmented into further segments, because our participants do not mention parts or regions of the *scissors*. MSFC also faces the same issue in cases where the algorithm clusters fixations falling on two unrelated regions of the image into one cluster. These observations strongly suggest that our framework would benefit from a segmentation technique that builds on both image features and gaze data.

**Figure 21.**
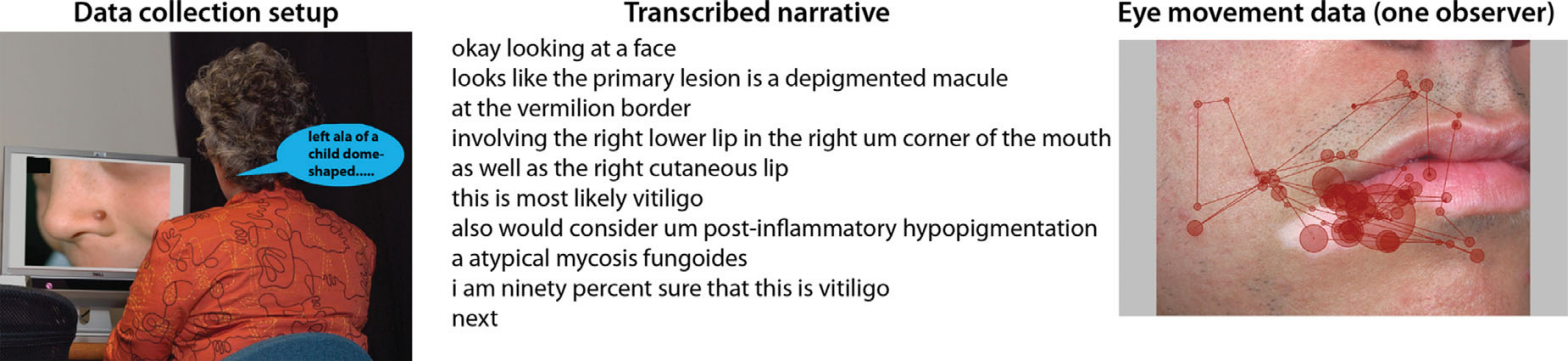
(*Left*) A dermatologist describing aloud the dermatology image to the experimenter while being eye tracked. (*Middle*) Example of transcribed narrative for the image shown in the right. (*Right*) Scanpath of an observer overlaid on the image. This figure has also appeared in Vaidyanathan et al. (2016).

We compared the framework's performance on the snag dataset containing general-domain images with the performance on the derm dataset containing images from the domain of dermatology (Vaidyanathan et al., 2016). This dataset consists of SNAG data for 26 dermatologists inspecting 29 dermatology images. Figure 21 shows our data set-up, an example of a transcribed narrative, and gaze data for this dataset. The data collection set-up was similar to the snag dataset. In this case, when using the adapted Master-Apprentice method, the experimenter functioned as an “apprentice” to elicit rich descriptions from the dermatologist. The dermatologists were instructed to “examine each image while moving toward a diagnosis and describe it aloud as if tutoring the experimenter.” The descriptions in this dataset usually included differential diagnosis, final diagnosis, and a self-estimated certainty of the final diagnosis. Again, dermatologists have specific shared terminology to refer to the morphology they describe; therefore, a manual annotation for this dataset was provided by an expert dermatologist using the RegionLabeler tool. More details regarding this dataset can be found in (Vaidyanathan et al., 2016). For the comparison, we only considered the results from alignment framework that used MSFC and *k*-means with *k* = 4. Interestingly, recall values are higher for the derm dataset when compared with the snag dataset. Recall values indicate the number of alignment pairs in the reference alignments that are also obtained in the framework's output alignments. One possible reason for high recall values could be that, as a result of task instructions, the derm dataset has a precise and limited vocabulary. Owing to the nature of the dermatology field, most of the regions in the images usually correspond with exactly one label. On the other hand, owing to the general-domain nature of the images in the snag dataset, many objects in the images correspond to various labels. For example, for the woman in the image in Figure 17, observers mentioned the labels *lady*, *woman*, and *female*. Thus, labels that were not mentioned by majority of the observers will have low probability of being associated with the corresponding image region leading to low recall values. Table 9 shows the average precision and recall values for the two datasets for the two segmentation methods.

**Table 9.** Comparison of precision and recall from the alignment framework for the two datasets for MSFC and *k*-means with *k* = 4. Precision is generally lower than recall except for the case of *k*-means with the snag dataset.

	MSFC		*k*-means	
	Precision	Recall	Precision	Recall
derm	0.45	0.56	0.41	0.56
snag	0.43	0.50	0.56	0.46

We also investigated the effect of the number of clusters obtained from MFSC on the framework's performance. Table 10 shows the Pearson's correlation coefficient between the number of clusters in the images and the precision, recall, and AER values. Also shown are the corresponding significance values. All three metrics are highly correlated with the number of clusters obtained using MSFC. The negative coefficient shows that as the number of clusters increases the performance decreases. This finding may be due to that, when there are fewer clusters, the output alignments are more likely to be right just by randomly guessing a cluster less number of clusters mean fewer incorrect output alignments. Further work is needed to investigate the cause of this correlation.

**Table 10.** Pearson's correlation value (*r*) and the corresponding significance value (*p*) between the performance metrics and the number of clusters obtained using MSFC.

	Precision	Recall	AER
snag	−0.29 (0.003)	−0.29 (0.003)	0.43 (5 × 10^−6^)

### Effect of parameters

**Figure 22.**
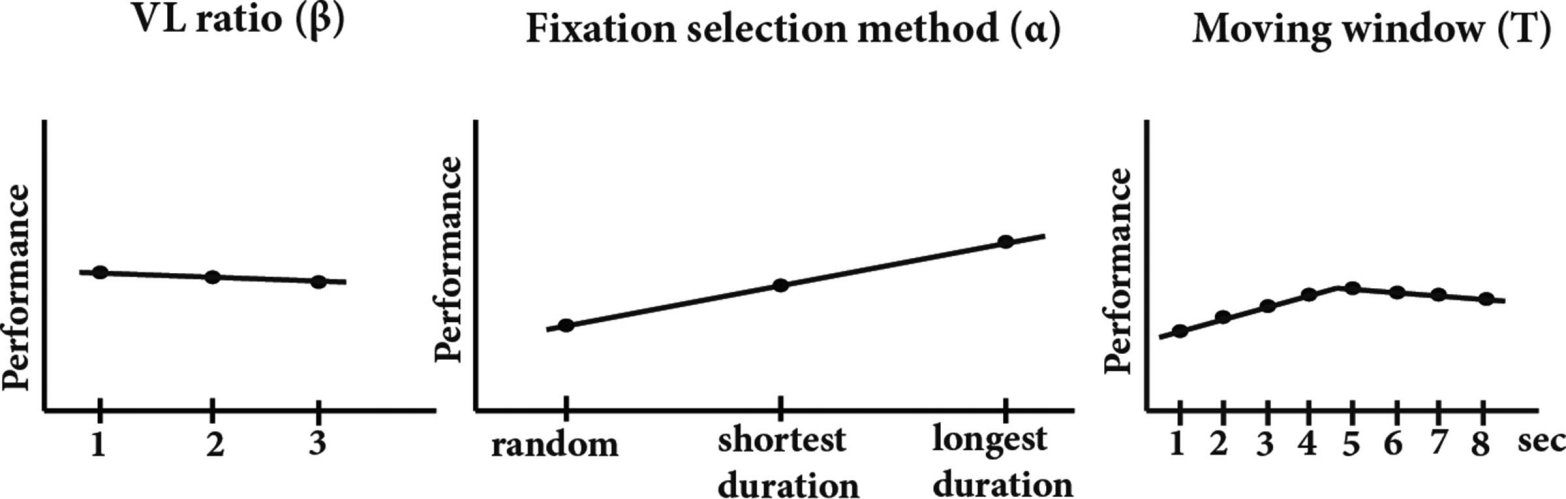
General effects on performance for (*Left*) VL ratio (β). (*Middle*) fixation selection method (α). (*Right*) moving window (*T*). The effect (positive or negative) reflected all measures. Default values used in this work resulting in high performance are: β = 1, α = longest duration, and *T* = 5 seconds.

As illustrated in Figure 22, we experimented with the following framework parameters: T, the sliding window, that aids in increasing training data size, β, the visual-linguistic ratio that ensures equal length of sequences of visual and linguistic units, and α, the method of visual unit selection referred to as fixation selection method. When the longest fixations within a sliding window were selected as visual units, the framework's performance was higher. This finding supports the intuitive notion that participants would fixate longer on image regions that play an important role in achieving the end goal. The default sliding window value of 5 seconds performs the best and higher values do not result in any improvement. Both the visual-linguistic ratio in our framework and the posterior decoding threshold in the Berkeley aligner have a negative effect on the framework's performance as they are increased. The observed trend was similar to the results for effect of parameters for the dataset involving dermatology images and experts (Vaidyanathan et al., 2015).

### Effect of manual correction versus ASR only

We manually corrected the transcriptions for 5 images and applied our annotation-alignment framework to the manually corrected narratives. Table 11 shows the performance of the framework with the corrected and uncorrected narratives. Narratives were on average 60 words in length and on average needed correction of three words resulting in an average word error rate of 5%. There is improvement in both precision and AER for all three clustering or segmentation methods between the uncorrected and corrected narratives. Using ASR transcriptions decreases manual labor by a substantial amount, but the performance improvement suggests the limitations of the automated transcription. Therefore, performance could be improved by using automated transcription followed by manual correction, which would require significantly less manual labor. The precision for corrected narratives is higher than for the uncorrected narratives. This could be due to higher percentage of overlap between linguistic units obtained from the corrected narratives and reference alignments. This finding indicates that we need improved methods to filter out or otherwise handle words that cannot be grounded in regions of the image.

**Table 11.** Comparison of average alignment performance across five images in the snag dataset for *uncorrected* versus *manually corrected* narratives. There is substantial improvement in both precision and AER for all the clustering or segmentation methods. The MSFC still offers the best AER performance for uncorrected versus corrected narratives.

	MSFC		*k*-means		GSEG	
	Uncorrected	Corrected	Uncorrected	Corrected	Uncorrected	Corrected
Precision	0.5	0.69	0.6	0.83	0.51	0.71
Recall	0.53	0.55	0.33	0.36	0.28	0.3
AER	0.48	0.37	0.55	0.47	0.62	0.55

## Future work and conclusions

Addressing RQ1 and RQ2, we have established quantitatively that people do not typically name-while-looking when speaking about visual content. Rather, they tend to fixate objects in images before they talk about them. We learn that, when humans describe a visual scene, there is almost always a time lag between fixations and words and that this lag is not fixed. Various quantitative and qualitative results reported in this work support RQ2 and highlight the usefulness of our multimodal snag dataset and the proposed framework (RQ3). Our snag dataset shows that observers on average spent approximately 0.58 seconds viewing the image before to the commencement of their description while dermatologists spent an average of 3 seconds inspecting the dermatology image before they began to talk (Vaidyanathan et al., 2013). This finding that observers might be trying to obtain a quick holistic view of the image and plan their speech before executing and that image complexity affects this timing. The snag dataset also shows that subject demographics (age, sex) do not seem to have an effect on the number of words and fixations people use to analyze a visual scene.

From our results, it is evident that the proposed alignment framework performs better than the simultaneous and delayed baselines. This finding shows that integration of multimodal data, specifically visual and linguistic data, is possible using bitext alignment. This conclusion is supported by both qualitative and quantitative results. The resulting annotations confirm that bitext alignment as used by our alignment framework can be used to obtain image region annotation. Additionally, the framework's performance also confirms that naturally elicited spoken narratives through the Master-Apprentice model (as opposed to written captions) are valuable for image region annotation. This framework does not depend on a specific type of expertise or image type and it can be applied to expert-domain images (Vaidyanathan et al., 2016) and images with different types of valence (Gangji et al., 2017; Haduong et al., 2018). Additionally, this framework can be extended to involve multiparty gaze and dialogue (Wang et al., 2019).

Overall, the MSFC clustering method outperforms the other segmentation methods. This finding indirectly validates the crucial role gaze data can play in an image region annotation framework. Other image segmentation methods such as *k*-means and GSEG provide comparable values of precision, suggesting that image features are also necessary for modeling image region annotation. Thus, to build an image annotation framework that can assist in developing advanced image-based application systems, we can leverage multimodal data elicited from humans. The ability of different segmentation methods to handle different aspects of images suggests that an extended framework could benefit by including an ensemble of distinct image segmentation techniques to address the heterogeneity of images and conceptual regions across images.

We observed that parameters such as the size of the time window used to expand the parallel corpus did not have major effect on the framework's performance, but that eliminating the sliding window entirely resulted in a degraded performance. Although there is an interaction between AER and the number of parallel sentences and their length, the most effective way to increase the size of the parallel corpus would likely be to collect data from additional observers rather than to adjust the parameters of the sliding window.

The framework's performance on uncorrected narratives suggests that there is potential in using automated speech-to-text transcription tools. However, the improved performance of the alignment framework on manually corrected narratives when compared to uncorrected narratives indicates that automated transcription followed by manual correction is advisable.

Currently, we are focused on extracting mostly nouns, adjectives, and some verbs as linguistic units, which consist of both units that can be grounded in an image and abstract units, but such abstract units cannot be aligned to any image region. In our future work, we will explore applying abstract concept filtering (Kiela et al., 2014) to remove these words from our linguistic units. Another method to remove linguistic units that are not present in the image from the narratives is by weighing linguistic units by the percent of participants that mention them. Our work somewhat achieves this by filtering out linguistic units that were only uttered once. The existing system could be improved further by incorporating a more holistic knowledge about the image and information about actions, verbs, and conceptual relations such as *meronymy*, commonly known as *part-whole* relationships, in both the linguistic and visual modalities.

Combining image features with gaze has the potential to further improve our results. Few prior studies have investigated gaze with image features for image annotation, but these studies either limit the end user's vocabulary or the region annotation to certain objects that can be detected using known computer vision techniques (Qu & Chai, 2008; Vasudevan et al., 2018a, 2018b). In our work, we instead innovatively integrate gaze and language to meaningfully annotate open domain images that are not limited by the type or number of objects. In contrast with standard computer vision techniques, gaze can capture which information in an image feature is useful at a particular point in time, for a particular perspective and semantic concept. This finding was confirmed by the preliminary results we obtained from applying our framework to the dermatology dataset. Our work acknowledges and highlights that gaze and speech data, in combination, provide comprehensive knowledge about the user's perception, thought processes, and intent. These advantages will eventually also benefit automated annotation frameworks that rely on image features.

MSFC and *k*-means with *k* = 4 show better performance than *k*-means with a larger *k* and GSEG owing to oversegmentation. Therefore, oversegmentation is an important issue to keep in mind when the new segmentation approach is designed for the framework. Apart from oversegmentation, images with more objects pose a challenge to the segmentation methods. For general-domain images such as ones discussed in this work, several state-of-the-art segmentation methods including deep learning methods have been shown to successfully perform on these images. We can further investigate the performance with DeepMask, a deep learning method and Convolutional Oriented Boundaries, a contour detection and hierarchical segmentation approach (Maninis et al., 2017). It would also be interesting to use deep learning methods with gaze and image features to identify improved visual units. Additionally, object recognition and scene understanding algorithms could help to group meaningful image regions and segments, eliminate spurious image regions, and provide more holistic interpretation. For example, in Figure 20 regardless of the segmentation algorithm used, an object recognition algorithm would identify the two individuals in the image as two people instead of several small segments.

A key advantage of our framework is its flexibility. For example, instead of using MSFC for detection of perceptually important regions for observers, one can apply k-means with RGB or other image features, GSEG, or any other segmentation or clustering method and different types of image features. Also, the approach is both domain independent and language dependent; it is generalizable to other contexts straightforwardly as long as simple part of speech tagging can be performed. In addition, we demonstrate its usefulness both in expert (Vaidyanathan et al., 2016) and in general domains by addressing the application to both visual environments. This flexibility of our framework makes it a particularly useful and powerful tool for integrating gaze and language.

The proposed alignment framework shows how we can adapt natural language processing and computer vision methods to creatively integrate visual and linguistic information. We show how such a multimodal integration could be used to achieve unsupervised semantic annotations for images. Like many datasets involving multimodal data elicitation from humans, our dataset is modest in size. Nevertheless, our results clarify our method's promise, and the quantitative metrics we apply and visualized results obtained support our conclusions. With advanced technologies such as virtual reality glasses, wearable eye-trackers, and smartglasses, collecting multimodal data could eventually become straightforward and natural resulting in more data that could benefit alignment-annotation framework and image-based application systems. Our work is an important contribution toward the highly challenging problem of fusing human-elicited multimodal data sources, a problem that will become increasingly important as such data become more common.

## Footnotes

1
https://mvrl-clasp.github.io/MultimodalFusionFramework/.

2
https://mvrl-clasp.github.io/SNAG/.

3
https://www.ibm.com/watson/services/speech-to-text/.

4 We empirically studied the impact of selecting visual units and the values of other parameters in different ways.

5
https://mvrl-clasp.github.io/SNAG/.

6
https://mvrl-clasp.github.io/MultimodalFusionFramework/.
